# Supplementary material for: Plasma proteins associated with cardiovascular death in patients with chronic coronary heart disease: A retrospective study
Source: PLoS Med. 2021 Jan 13;18(1):e1003513. doi: 10.1371/journal.pmed.1003513 (PMC7817029; doi:10.1371/journal.pmed.1003513)
Supplement: S1 Text — (PDF) [file pmed.1003513.s002.pdf]

Statistical Analysis Plan

**Study Title:**	STABILITY PEA
**Biostatistician:**	Niclas Eriksson, UCR
**Clinical Data Manager:**	Niclas Eriksson, UCR
**Principal investigator:**	Lars Wallentin, UCR<br>Agneta Siegbahn, UCR
**Sponsor representatitive:**	N/A
**SAP version:**	1.1

Revised version March 20, 2017
Added deviations from SAP October 19, 2020

Table of Contents

1 Introduction 3
2 Abbreviations 3
3 Study Objectives and Endpoints 4
3.1 Objectives 4
3.2 Endpoints 4
4 Study design 4
4.1 Definition of Analysis Populations 5
4.2 Analysis considerations based on the study design 5
5 Description of statistical analysis 5
5.1 Demographics and baseline characteristics 5
5.1.1 Demographics 5
5.1.2 Biochemical biomarker analyses 6
5.1.3 PEA biomarkers description and associations between biomarkers 6
5.2 Statistical analyses of associations to outcomes 6
5.2.1 Univariate analyses 6
5.2.2 Multiple adjusted analyses 7
5.2.3 Random forest analyses 7
5.2.4 Multiple analyses of biomarkers of importance 7
6 Determination of sample size 7
7 Deviations from the SAP v1.1 7
8 References 8

1 Introduction

The purpose of this plan is to outline the details of the statistical analyses to be performed in the STABILITY PEA project.

2 Abbreviations

ACE	Angiotensin-converting enzyme
AII	Angiotensin II receptor
CABG	Coronary artery bypass graft
CAD	Coronary artery disease
CHD	Coronary heart disease
CRP	C-reactive protein
CVD	Cardiovascular disease
eGFR	Estimated Glomerular Filtration Rate
GDF-15	Growth differentiation factor 15
HDL	High-density lipoprotein
HF	Heart failure
IL-6	Interleukin-6
LDL	Low-density lipoprotein
Lp-PLA$_2$	Lipoprotein-associated Phospholipase A2
MACE	Major adverse cardiac events
MCE	Major coronary events
MI	Myocardial infarction
NTproBNP	N-terminal prohormone of brain natriuretic peptide
PAD	Peripheral artery disease
PCI	Percutaneous coronary intervention
PEA	Proximity Extension Assay

SAP	Statistical analysis plan
TnT	Troponin T

3 Study Objectives and Endpoints

3.1 Objectives

The primary aim of this explorative study is to investigate which biomarkers and/or clusters of biomarkers in the OLINK PEA panels CVD I and Inflammation are associated with cardiovascular events in patients with stable coronary artery disease (CAD) in the STABILITY trial. The analyses are based on previous studies[1, 2] that have established a strong prognostic value for biomarkers of renal dysfunction (Cystatin-C or eGFR), cardiac dysfunction (NTproBNP and troponin) as well as inflammation (GDF-15, IL-6) when determining cardiovascular outcomes in patients with stable CAD. In addition, many proteins are renally excreted and their levels affected by variations in renal function. In order to elucidate the value of a new biomarker it is therefore necessary to investigate and adjust for the impact of established indicators of renal and cardiac dysfunction and eventually also inflammation. Finally, from a clinical perspective a biomarker is only important if it provides incremental prognostic information beyond available clinical characteristics.

3.2 Endpoints

- MACE (major adverse cardiac event, i.e., cardiovascular death, myocardial infarction (MI), stroke)
- MCE (major coronary events, i.e., coronary death, MI, urgent coronary revascularization)
- HF (heart failure)
- CVD (cardiovascular death)
- MI
- CVD or HF

4 Study design

The PEA sample is based on an unstratified case-cohort design based on the Full cohort, defined as STABILITY patients with biomarker data on all of Troponin T, NTproBNP, GDF-15 and $LpPLA_2$, constituting N=14124 patients.
The PEA sample consists of:

1. All patients that experienced adjudicated MACE (N=1403) or adjudicated hospitalization for heart failure (N=305) during the follow-up time for cardiovascular events (total N= 1588).
2. A subcohort randomly sampled without replacement from the Full cohort (including cases) (N=3115). Each Full cohort patient was selected with a probability of 3115/14124. The size of the subcohort was chosen to give approximately 2 non-cases per case.

The subcohort includes 330 cases, and the total PEA sample size is N=4373.

4.1 Definition of Analysis Populations

The analyses will be performed based on the different endpoints as outlined above. For each individual or composite endpoint the sample will contain the random subcohort and, in addition, all cases with the investigated endpoint.

4.2 Analysis considerations based on the study design

It is anticipated that the data will be analysed using appropriately sampling probability weighted Cox regression with an appropriate variance-covariance estimation method. Similar appropriate methods will be used to obtain any other descriptive and inferential statistics. Since the total amount of data is large and the subcohort constitutes as much as 20% of the original cohort, the different popular weighting schemes and covariance estimation methods can be assumed to give similar results, hence the choice will be based on practical considerations. Other methods may be applied, including methods incorporating Full cohort data. It can be noted that the Full sample can be regarded as already enriched with cases through the inclusion criteria.

5 Description of statistical analysis

The following chapters will apply to all endpoints.

5.1 Demographics and baseline characteristics

5.1.1 Demographics

The baseline demographics will be tabulated in total and by endpoint (and those indicated with asterisk will be used for adjustment for baseline characteristics in the analyses)

- Age*
- Sex*
- BMI*, weight, height
- Current smoking*
- Hypertension*
- Diabetes*
- Previous MI*
- Previous coronary revascularization (CABG or PCI)
- Previous stroke*
- Previous peripheral artery disease (PAD)*
- Multivessel coronary heart disease (CHD)
- Polyvascular disease
- Randomized treatment*
- Medications at randomization
    - Aspirin
    - Clopidogrel
    - Beta-blockers
    - Statin
    - ACE or AII inhibition
    - Insulin

- Oral antidiabetic

5.1.2 Biochemical biomarker analyses

Biomarkers (other than PEA) will be tabulated (in total and by endpoint) and those indicated with asterisk will be used for adjustment in some analyses.

- Cystatin-C*
- eGFR
- NTproBNP*
- TnT*
- CRP
- IL-6*
- GDF-15*
- LDL-cholesterol
- HDL-cholesterol
- Triglycerides
- HbA1c

(Lp-PLA_2 will not be included in the biomarker evaluation because of a complex interaction with randomized treatment that might not be compensated for by the adjustments. The Lp-PLA_2 results have already been published as a separate manuscript[3].

5.1.3 PEA biomarkers description and associations between biomarkers

Biomarkers on the PEA chips CVD I and Inflammation will be tabulated in total and by endpoint.

The correlation between the established biomarkers as listed above and the PEA biomarkers will be presented as a table with correlation coefficients (*p*-values illustrated by stars) with the established biomarkers in the same order as above in horizontal direction and then the same markers followed by the PEA markers in the vertical direction.

The above correlation table will also be presented in a reduced format excluding all lines of biomarkers with no correlation coefficient > 0.29.

5.2 Statistical analyses of associations to outcomes

5.2.1 Univariate analyses

Cox-regression analyses will be performed for each biomarker on the PEA chip and for the additional biomarkers listed under chapter 5.1.2. Results will be presented in a table as well as in a forest plot.

The correlation analyses, as outlined in 6.1.3, will be performed including the established biomarkers and only the PEA-biomarkers with statistically significant associations with the investigated outcome. An attempt will be performed to identify clusters of biomarkers with similar associations with both established biomarkers and outcomes, suggesting them being indicators of a similar underlying process.

5.2.2 Multiple adjusted analyses

Same Cox regression analysis as in chapter 5.2.1 with adjustment in the following steps

1. baseline characteristics indicated with asterisk in 5.1.1.
2. add also cystatin-C
3. add also NTproBNP and TnT
4. add also GDF-15 and IL-6

Biomarkers on the PEA chip that were also measuring the adjustment biomarker will not be included in these analyses. Likewise only one PEA result for each biomarker will be included (if the PEA marker is measured on more than one panel).

5.2.3 Random forest analyses

Two random forest analyses will be performed:

1. Including all PEA variables
2. Including PEA variables as well as demographics and other biomarkers

Biomarkers on the PEA chip that were also measured by an established biomarker method will not be included in these analyses. Likewise only one PEA result for each biomarker will be included.

5.2.4 Multiple analyses of biomarkers of importance

Given the results of previous chapters, additional multiple Cox-regression analyses may be performed, investigating the predictive performance, for:

1. A model including previously known baseline covariates known to affect each outcome (taken from prior projects)
2. In addition to 1, previously known biomarkers known to affect each outcome (taken from prior projects)
3. In addition to 1 and 2, add PEA markers of importance

If PEA markers improve the discrimination of certain outcomes beyond established markers the discriminative value may also be investigated by c-index and other appropriate measures.

6 Determination of sample size

See chapter 4.

7 Deviations from the SAP v1.1

1. All Cox-regressions include the sampling weights (according to the design) and variance estimates are calculated using a robust sandwich estimator
2. Boruta analyses[4] were performed in addition to the random forest analyses
3. LP-PLA_2 was not excluded from the analyses

8 References

1. Correa S, Morrow DA, Braunwald E, Davies RY, Goodrich EL, Murphy SA, et al. Cystatin C for Risk Stratification in Patients After an Acute Coronary Syndrome. J Am Heart Assoc. 2018;7:e009077.
2. Hagstrom E, James SK, Bertilsson M, Becker RC, Himmelmann A, Husted S, et al. Growth differentiation factor-15 level predicts major bleeding and cardiovascular events in patients with acute coronary syndromes: results from the PLATO study. Eur Heart J. 2016;37:1325-33.
3. Wallentin L, Held C, Armstrong PW, Cannon CP, Davies RY, Granger CB, et al. Lipoprotein-Associated Phospholipase A2 Activity Is a Marker of Risk But Not a Useful Target for Treatment in Patients With Stable Coronary Heart Disease. J Am Heart Assoc. 2016;5:e003407.
4. Kursa MB, Rudnicki WR. Feature Selection with the Boruta Package. J Stat Softw. 2010;36:1-13.
