# Supplementary material for: Plasma proteins associated with cardiovascular death in patients with chronic coronary heart disease: A retrospective study
Source: PLoS Med. 2021 Jan 13;18(1):e1003513. doi: 10.1371/journal.pmed.1003513 (PMC7817029; doi:10.1371/journal.pmed.1003513)
Supplement: S1 Table — (PDF) [file pmed.1003513.s003.pdf]

Abbreviation	Protein name	UniProt No	Olink panel
4E-BP1	Eukaryotic translation initiation factor 4E-binding protein 1	Q13541	Inflammation
ADA	Adenosine Deaminase	P00813	Inflammation
AGRP	Agouti-related protein	O00253	CVD I
AM	Adrenomedullin	P35318	CVD I
ARTN	Artemin	Q5T4W7	Inflammation
AXIN1	Axin-1	O15169	Inflammation
BDNF	Brain-derived neurotrophic factor	P23560	Inflammation
Beta-NGF	Beta-nerve growth factor	P01138	CVD I*
BNP	Natriuretic peptides B	P16860	CVD I
CA-125	Ovarian cancer-related tumor marker 125	Q8WXI7	CVD I
CASP-8	Caspase-8	Q14790	CVD I*
CCL11	Eotaxin-1	P51671	Inflammation
CCL19	C-C motif chemokine 19	Q99731	Inflammation
CCL20	C-C motif chemokine 20	P78556	CVD I*
CCL23	C-C motif chemokine 23	P55773	Inflammation
CCL25	C-C motif chemokine 25	O15444	Inflammation
CCL28	C-C motif chemokine 28	Q9NRJ3	Inflammation
CCL3	C-C motif chemokine 3	P10147	CVD I
CCL4	C-C motif chemokine 4	P13236	CVD I*
CD244	Natural killer cell receptor 2B4	Q9BZW8	Inflammation
CD40	Tumor necrosis factor receptor superfamily member 5	P25942	CVD I*
CD40L	CD40 ligand	P29965	CVD I
CD5	T-cell surface glycoprotein CD5	P06127	Inflammation
CD6	T cell surface glycoprotein CD6 isoform	Q8WWJ7	Inflammation
CDCP1	CUB domain-containing protein 1	Q9H5V8	Inflammation
CHI3L1	Chitinase-3-like protein 1	P36222	CVD I
CSF-1	Macrophage colony-stimulating factor 1	P09603	CVD I*
CST5	Cystatin D	P28325	Inflammation
CSTB	Cystatin-B	P04080	CVD I
CTSD	Cathepsin D	P07339	CVD I
CTSL1	Cathepsin L1	P07711	CVD I
CX3CL1	Fractalkine	P78423	CVD I*
CXCL1	C-X-C motif chemokine 1	P09341	CVD I*
CXCL10	C-X-C motif chemokine 10	P02778	Inflammation
CXCL11	C-X-C motif chemokine 11	O14625	Inflammation
CXCL16	C-X-C motif chemokine 16	Q9H2A7	CVD I
CXCL5	C-X-C motif chemokine 5	P42830	Inflammation
CXCL6	C-X-C motif chemokine 6	P80162	CVD I*
CXCL9	C-X-C motif chemokine 9	Q07325	Inflammation
Dkk-1	Dickkopf-related protein 1	O94907	CVD I
DNER	Delta and Notch-like epidermal growth factor-related recep	Q8NFT8	Inflammation

Abbreviation	Protein name	UniProt No	Olink panel
ECP	Eosinophil cationic protein	P12724	CVD I
EGF	Epidermal growth factor	P01133	CVD I
EN-RAGE	Protein S100-A12	P80511	CVD I*
ESM-1	Endothelial cell-specific molecule 1	Q9NQ30	CVD I
FABP4	Fatty acid-binding protein, adipocyte	P15090	CVD I
FAS	Tumor necrosis factor receptor superfamily member 6	P25445	CVD I
FGF-19	Fibroblast growth factor 19	O95750	Inflammation
FGF-21	Fibroblast growth factor 21	Q9NSA1	Inflammation
FGF-23	Fibroblast growth factor 23	Q9GZV9	CVD I*
FGF-5	Fibroblast growth factor 5	Q8NF90	Inflammation
Flt3L	Fms-related tyrosine kinase 3 ligand	P49771	Inflammation
FS	Follistatin	P19883	CVD I
GAL	Galanin peptides	P22466	CVD I
Gal-3	Galectin-3	P17931	CVD I
GDF-15	Growth/differentiation factor 15	Q99988	CVD I
GH	Growth hormone	P01241	CVD I
HB-EGF	Heparin-binding EGF-like growth factor	Q99075	CVD I
hGDNF	Glial cell line-derived neurotrophic factor	P39905	Inflammation
HGF	Hepatocyte growth factor	P14210	CVD I*
hk11	Kallikrein-11	Q9UBX7	CVD I
HSP 27	Heat shock 27 kDa protein	P04792	CVD I
IFN-gamma	Interferon gamma	P01579	Inflammation
IL-1 alpha	Interleukin-1 alpha	P01583	Inflammation
IL-10	Interleukin-10	P22301	Inflammation
IL-10RA	Interleukin-10 receptor subunit alpha	Q13651	Inflammation
IL-10RB	Interleukin-10 receptor subunit beta	Q08334	Inflammation
IL-12B	Interleukin-12 subunit beta	P29460	Inflammation
IL-13	Interleukin-13	P35225	Inflammation
IL-15RA	Interleukin-15 receptor subunit alpha	Q13261	Inflammation
IL-16	Interleukin-16	Q14005	CVD I
IL-17A	Interleukin-17A	Q16552	Inflammation
IL-17C	Interleukin-17C	Q9P0M4	Inflammation
IL-18	Interleukin-18	Q14116	CVD I*
IL-18R1	Interleukin-18 receptor 1	Q13478	Inflammation
IL-1ra	Interleukin-1 receptor antagonist protein	P18510	CVD I
IL-2	Interleukin-2	P60568	Inflammation
IL-20	Interleukin-20	Q9NYY1	Inflammation
IL-20RA	Interleukin-20 receptor subunit alpha	Q9UHF4	Inflammation
IL-22 RA1	Interleukin-22 receptor subunit alpha-1	Q8N6P7	Inflammation
IL-24	Interleukin-24	Q13007	Inflammation
IL-27	Interleukin-27	Q8NEV9<br>Q14213	CVD I
IL-2RB	Interleukin-2 receptor subunit beta	P14784	Inflammation

Abbreviation	Protein name	UniProt No	Olink panel
IL-33	Interleukin-33	O95760	Inflammation
IL-4	Interleukin-4	P05112	CVD I*
IL-5	Interleukin-5	P05113	Inflammation
IL-6	Interleukin-6	P05231	CVD I*
IL-6RA	Interleukin-6 receptor subunit alpha	P08887	CVD I
IL-7	Interleukin-7	P13232	Inflammation
IL-8	Interleukin-8	P10145	CVD I*
ITGB1BP2	Melusin	Q9UKP3	CVD I
KLK6	Kallikrein-6	Q92876	CVD I
LAP TGF-beta-1	Latency-associated peptide transforming growth factor beta 1	P01137	Inflammation
LEP	Leptin	P41159	CVD I
LIF	Leukemia inhibitory factor	P15018	Inflammation
LIF-R	Leukemia inhibitory factor receptor	P42702	Inflammation
LOX-1	Lectin-like oxidized LDL receptor 1	P78380	CVD I
mAmP	Membrane-bound aminopeptidase P	O43895	CVD I
MB	Myoglobin	P02144	CVD I
MCP	Monocyte chemotactic protein 1	P13500	CVD I*
MCP-2	Monocyte chemotactic protein 2	P80075	Inflammation
MCP-3	Monocyte chemotactic protein 3	P80098	Inflammation
MCP-4	Monocyte chemotactic protein 4	Q99616	Inflammation
MIP-1 alpha	Macrophage inflammatory protein 1-alpha	P10147	Inflammation
MMP-1	Matrix metalloproteinase-1	P03956	CVD I*
MMP-10	Matrix metalloproteinase-10	P09238	CVD I*
MMP-12	Matrix metalloproteinase-12	P39900	CVD I
MMP-3	Matrix metalloproteinase-3	P08254	CVD I
MMP-7	Matrix metalloproteinase-7	P09237	CVD I
MPO	Myeloperoxidase	P05164	CVD I
NEMO	NF-kappa-B essential modulator	Q9Y6K9	CVD I
NRTN	Neurturin	Q99748	Inflammation
NT-3	Neurotrophin-3	P20783	Inflammation
NT-pro- BNP	N-terminal pro-B-type natriuretic peptide	NR	CVD I
OPG	Osteoprotegerin	O00300	CVD I*
OSM	Oncostatin-M	P13725	Inflammation
PAPPA	Pappalysin-1	Q13219	CVD I
PAR-1	Proteinase-activated receptor 1	P25116	CVD I
PDGF subunit B	Platelet-derived growth factor subunit B	P01127	CVD I
PD-L1	Programmed cell death 1 ligand 1	Q9NZQ7	Inflammation
PECAM-1	Platelet endothelial celll adhesion molecule	P16284	CVD I
PlGF	Placenta growth factor	P49763	CVD I
PRL	Prolactin	P01236	CVD I
PSGL-1	P-selectin glycoprotein ligand 1	Q14242	CVD I
PTX3	Pentraxin-related protein PTX3	P26022	CVD I

Abbreviation	Protein name	UniProt No	Olink panel
RAGE	Receptor for advanced glycosylation end products	Q15109	CVD I
REN	Renin	P00797	CVD I
RETN	Resistin	Q9HD89	CVD I
SCF	Stem cell factor	P21583	CVD I*
SELE	E-selectin	P16581	CVD I
SIRT2	SIR2-like protein	Q8IXJ6	CVD I*
SLAMF1	Signaling lymphocytic activation molecule	Q13291	Inflammation
SPON1	Spondin-1	Q9HCB6	CVD I
SRC	Proto-oncogene tyrosine-protein kinase Src	P12931	CVD I
ST1A1	Sulfotransferase 1A1	P50225	Inflammation
ST2	ST2 protein	Q01638	CVD I
STAMPB	STAM-binding protein	O95630	Inflammation
TF	Tissue factor	P13726	CVD I
TGF-alpha	Transforming growth factor alpha	P01135	Inflammation
TIE2	Angiopoietin-1 receptor	Q02763	CVD I
TIM	TIM-1	Q96D42	CVD I
TM	Thrombomodulin	P07204	CVD I
TNF	Tumor necrosis factor	P01375	Inflammation
TNFB	TNF-beta	P01374	Inflammation
TNF-R1	Tumor necrosis factor receptor 1	P19438	CVD I
TNF-R2	Tumor necrosis factor receptor 2	P20333	CVD I
TNFRSF9	Tumor necrosis factor receptor superfamily member 9	Q07011	Inflammation
TNFSF14	Tumor necrosis factor ligand superfamily member 14	O43557	CVD I*
t-PA	Tissue-type plasminogen activator	P00750	CVD I
TRAIL	TNF-related apoptosis-inducing ligand	P50591	CVD I*
TRAIL-R2	TNF-related apoptosis-inducing ligand receptor 2	O14763	CVD I
TRANCE	TNF-related activation-induced cytokine	O14788	CVD I*
TSLP	Thymic stromal lymphopoietin	Q969D9	Inflammation
TWEAK	Tumor necrosis factor (Ligand) superfamily, member 12	Q4ACW9	Inflammation
uPA	Urokinase-type plasminogen activator	P00749	Inflammation
U-PAR	Urokinase plasminogen activator surface receptor	Q03405	CVD I
VEGF-A	Vascular endothelial growth factor A	P15692	CVD I*
VEGF-D	Vascular endothelial growth factor D	O43915	CVD I

*Proteins were included on both panels, NPX values from CVD I panel were used.
