# Supplementary material for: Plasma proteins associated with cardiovascular death in patients with chronic coronary heart disease: A retrospective study
Source: PLoS Med. 2021 Jan 13;18(1):e1003513. doi: 10.1371/journal.pmed.1003513 (PMC7817029; doi:10.1371/journal.pmed.1003513)
Supplement: S2 Table — (A) Data availability in all 4,127 patients in the STABILITY cohort. (B) Data availability in all 1,331 patients in the LURIC cohort. LURIC, Ludwigshafen Risk and Cardiovascular Health; NPX, normalized protein expression; PEA, proximity extension assay; STABILITY, STabilization of Atherosclerotic plaque By Initiation of darapLadIb TherapY. (PDF) [file pmed.1003513.s004.pdf]

A

Biomarker	% values below lower limit of detection	Comment
BDNF	100.0	Removed from further analyses
IL-2	99.9	Removed from further analyses
IL-22-RA1	99.7	Removed from further analyses
TSLP	99.5	
IFN-gamma	99.2	
IL33	98.7	
IL-1-alpha	97.5	
IL-2RB	97.4	
ARTN	96.9	
IL-4	96.7	
NRTN	95.7	
IL-20	95.5	
IL-24	94.5	
IL-20RA	94.3	
IL13	93.7	
LIF	93.3	
TNF	89.8	
IL-10RA	84.3	
IL-5	83.6	
ITGB1BP2	78.9	
IL-1ra	67.4	
IL-17C	64.7	
IL-17A	61.6	
AXIN1	54.7	
mAmP	41.2	
BNP	39.3	
FGF-5	34.3	
Beta-NGF	18.8	
MCP-3	19.0	
ST1A1	18.0	
IL-15RA	15.9	
SIRT2	12.4	
EN-RAGE	7.9	
CCL28	6.8	
MMP-7	7.6	
CD40-L	7.5	
NEMO	6.7	
GDNF	5.0	
OSM	4.3	
PSGL-1	0.2	
NT-3	2.4	
CASP-8	3.0	
HSP-27	2.9	

Biomarker	% values below lower limit of detection	Comment
NT-pro-BNP	2.6	
TRANCE	2.4	
IL7	2.2	
hK11	1.2	
PAPPA	1.6	
CXCL16	0.1	
MPO	0.0	
CSF-1	0.0	
DNER	0.0	
LIF-R	0.1	
TGF-alpha	0.1	
TIE2	0.0	
SPON1	0.0	
CD244	0.0	
IL10	0.4	
PECAM-1	0.0	
AM	1.2	
TM	0.0	
TWEAK	0.0	
ADA	0.1	
TRAIL	0.0	
U-PAR	0.0	
TF	0.0	
OPG	0.0	
PD-L1	0.1	
PlGF	0.0	
CD5	0.0	
FABP4	0.6	
CA-125	0.8	
IL-18R1	0.0	
ECP	0.9	
MCP-1	0.0	
FAS	0.0	
IL27-A	0.0	
TNFB	0.1	
ESM-1	0.0	
SCF	0.0	
TNF-R1	0.0	
CTSL1	0.0	
Gal-3	0.0	
CCL23	0.0	
uPA	0.0	
FS	0.0	
HGF	0.0	

Biomarker	% values below lower limit of detection	Comment
IL-10RB	0.0	
KLK6	0.0	
TNF-R2	0.0	
CCL11	0.0	
CD6	0.0	
CST5	0.0	
IL-16	0.0	
VEGF-A	0.0	
VEGF-D	0.1	
CD40	0.0	
Flt3L	0.0	
CX3CL1	0.0	
SLAMF1	0.1	
t-PA	0.0	
CTSD	0.0	
MMP-10	0.0	
AGRP	0.1	
CCL3	0.0	
HB-EGF	0.0	
RETN	0.0	
IL-6RA	0.0	
MB	0.0	
IL-18	0.0	
IL-12B	0.0	
RAGE	0.0	
CCL4	0.0	
IL-8	0.0	
TRAIL-R2	0.0	
MMP-1	0.4	
TNFRSF9	0.0	
CCL25	0.0	
CDCP1	0.0	
MCP-2	0.0	
LAP-TGF-beta-1	0.0	
PTX3	0.1	
SELE	0.0	
CSTB	0.0	
CXCL11	0.0	
CXCL9	0.0	
EGF	0.6	
PAR-1	0.0	
TNFSF14	0.0	
MCP-4	0.0	
FGF-23	0.0	

Biomarker	% values below lower limit of detection	Comment
LEP	0.4	
PDGF-Subunit-B	0.6	
PRL	0.2	
ST2	0.0	
4E-BP1	0.1	
MMP-3	0.0	
CXCL10	0.0	
LOX-1	0.0	
CCL19	0.0	
FGF-21	0.1	
Dkk-1	0.0	
GH	0.3	
GDF-15	0.0	
GAL	0.0	
STAMPB	0.0	
IL-6	0.0	
MMP-12	0.0	
FGF-19	0.0	
REN	0.0	
TIM	0.0	
CHI3L1	0.1	
CCL20	0.0	
CXCL5	0.0	
SRC	0.1	
CXCL1	0.0	
CXCL6	0.0	

B

Biomarker	% below lower limit of detection	Comment
ITGB1BP2	99.8	Removed from further analyses
IL-4	99.3	Removed from further analyses (Cox-analyses fail)
PAPPA	98.6	
PSGL-1	97.1	
CD40-L	90.6	
PTX3	88.1	
FS	85.2	
BNP	70.5	
CA-125	67.5	
Beta-NGF	66.1	
MMP-1	62.3	
mAmP	55.3	
HSP-27	25.7	
CASP-8	23.5	
SIRT2	22.9	
NT-pro-BNP	21.6	
IL-6	8.6	
PRL	6.8	
TNFSF14	6.7	
IL-16	6.5	
TRANCE	4.8	
SPON1	2.6	
NEMO	1.9	
PDGF-Subunit-B	1.8	
GH	1.5	
FABP4	0.6	
CSTB	0.5	
ECP	0.5	
ESM-1	0.3	
IL-1ra	0.3	
CXCL16	0.2	
EN-RAGE	0.2	
LEP	0.2	
REN	0.2	
CHI3L1	0.1	
Gal-3	0.1	
IL27-A	0.1	
MMP-3	0.1	
AGRP	0	
AM	0	
CCL20	0	
CCL3	0	

Biomarker	% below lower limit of detection	Comment
CCL4	0	
CD40	0	
CSF-1	0	
CTSD	0	
CTSL1	0	
CX3CL1	0	
CXCL1	0	
CXCL6	0	
Dkk-1	0	
EGF	0	
FAS	0	
FGF-23	0	
GAL	0	
GDF-15	0	
HB-EGF	0	
HGF	0	
hK11	0	
IL-18	0	
IL-6RA	0	
IL-8	0	
KLK6	0	
LOX-1	0	
MB	0	
MCP-1	0	
MMP-10	0	
MMP-12	0	
MMP-7	0	
MPO	0	
OPG	0	
PAR-1	0	
PECAM-1	0	
PlGF	0	
RAGE	0	
RETN	0	
SCF	0	
SELE	0	
SRC	0	
ST2	0	
t-PA	0	
TF	0	
TIE2	0	
TIM	0	
TM	0	
TNF-R1	0	

Biomarker	% below lower limit of detection	Comment
TNF-R2	0	
TRAIL	0	
TRAIL-R2	0	
U-PAR	0	
VEGF-A	0	
VEGF-D	0	
