# Supplementary material for: Plasma proteins associated with cardiovascular death in patients with chronic coronary heart disease: A retrospective study
Source: PLoS Med. 2021 Jan 13;18(1):e1003513. doi: 10.1371/journal.pmed.1003513 (PMC7817029; doi:10.1371/journal.pmed.1003513)
Supplement: S3 Table — STABILITY, STabilization of Atherosclerotic plaque By Initiation of darapLadIb TherapY. (PDF) [file pmed.1003513.s005.pdf]

**Baseline characteristics**	**Subcohort (N=2932)**	**All patients (N=15828)**
Age (years)	59/65/71	59/65/71
Sex: Male	82% (2397)	81% (12861)
Body mass index (kg/m^2)	25.5/28.1/31.6	25.5/28.3/31.7
Body weight (kg)	71.8/82.0/93.6	71.6/82.0/94.0
Smoker or prior smoker	20% (580)	20% (3228)
Hypertension	70% (2058)	72% (11318)
Diabetes	39% (1134)	39% (6136)
Prior myocardial infarction	59% (1737)	59% (9323)
Prior PCI or CABG	75% (2207)	75% (11863)
Prior stroke or TIA	9% (255)	9% (1349)
Prior PAD	8% (233)	8% (1339)
Prior multivessel CHD	14% (401)	15% (2390)
Polyvascular disease	15% (442)	15% (2372)
Randomized to darapladib	51% (1509)	50% (7924)
**Medications at baseline**		
Aspirin	92% (2704)	92% (14623)
P_2Y_{12} -inhibitors	34% (986)	34% (5403)
Beta-blockers	79% (2315)	79% (12508)
Statin treatment	97% (2852)	97% (15398)
ACE- or angiotensin receptor inhibitors	76% (2234)	77% (12201)

Categorical variables are reported as % (n) whereas continuous are reported by the percentiles 25th/50th/75th. PCI, percutaneous coronary intervention; CABG, coronary artery bypass grafting; TIA, transient ischemic attack; PAD, peripheral artery disease; CHD, coronary heart disease; ACE, angiotensin-converting enzyme.
