# Supplementary material for: Plasma proteins associated with cardiovascular death in patients with chronic coronary heart disease: A retrospective study
Source: PLoS Med. 2021 Jan 13;18(1):e1003513. doi: 10.1371/journal.pmed.1003513 (PMC7817029; doi:10.1371/journal.pmed.1003513)
Supplement: S4 Table — (A) Reproducibility of biomarkers determined in both the CVD1 and Inflammation OLINK PEA panels. (B) Comparisons between biomarkers associations with CV death when using measurements with conventional (lab log2) and PEA assays (NPX) as evaluated by univariate Cox regression analyses. CV, cardiovascular; NPX, normalized protein expression; PEA, proximity extension assay. (PDF) [file pmed.1003513.s006.pdf]

A

	Difference				
**Biomarker**	**Mean**	**SD**	**Lower 95% limit of agreement**	**Upper 95% limit of agreement**	**Correlation (Spearman)**
Beta-NGF	-0,82	0,53	-1,86	0,21	0,62
CASP-8	-0,79	0,4	-1,58	0	0,93
CCL20	-3,38	0,28	-3,94	-2,82	0,97
CCL3	-1,56	0,23	-2,01	-1,12	0,94
CCL4	-2,9	0,57	-4,02	-1,79	0,78
CD40	-0,97	0,24	-1,43	-0,5	0,88
CSF-1	-3,18	0,18	-3,54	-2,82	0,77
CX3CL1	-1,11	0,24	-1,58	-0,65	0,87
CXCL1	-0,75	0,37	-1,48	-0,02	0,96
CXCL6	-1,54	0,32	-2,16	-0,92	0,96
EN-RAGE	-0,89	0,45	-1,77	-0,01	0,9
FGF-23	-0,11	0,26	-0,62	0,4	0,95
HGF	-0,9	0,22	-1,32	-0,47	0,88
IL-18	-4,79	0,23	-5,24	-4,33	0,93
IL-4	-0,79	0,59	-1,95	0,38	0,61
IL-6	-1,22	0,24	-1,7	-0,74	0,97
IL-8	-0,7	0,27	-1,22	-0,17	0,96
MCP-1	-0,5	0,22	-0,92	-0,07	0,9
MMP-1	2,35	0,36	1,64	3,05	0,95
MMP-10	-0,03	0,26	-0,54	0,48	0,91
OPG	-1,09	0,2	-1,49	-0,7	0,87
SCF	0,07	0,18	-0,28	0,43	0,93
SIRT2	-0,02	0,68	-1,36	1,31	0,94
TNFSF14	-1,4	0,24	-1,87	-0,92	0,93
TRAIL	-2,57	0,18	-2,92	-2,22	0,86
TRANCE	-0,76	0,44	-1,63	0,1	0,82
VEGF-A	-0,45	0,27	-0,97	0,08	0,85

B

**Protein**	**SD**	**HR (95% CI)**	**C-index**	**P-value**
NTproBNP (lab log2)	1.74	2.94 (2.65-3.27)	0.769	2.52e-88
NTproBNP (NPX)	1.21	3.16 (2.57-3.87)	0.718	3.25e-28
GDF-15 (lab log2	0.79	1.88 (1.72-2.05)	0.675	1.49e-44
GDF-15 (NPX)	0.68	1.94 (1.77-2.13	0.677	3.72e-45
IL-6 (lab log2)	0.95	1.48 (1.38-1.59)	0.650	6.54e-27
IL-6 (NPX)	0.95	1.65 (1.52-1.79)	0.654	1.10e-34
