# Supplementary material for: Plasma proteins associated with cardiovascular death in patients with chronic coronary heart disease: A retrospective study
Source: PLoS Med. 2021 Jan 13;18(1):e1003513. doi: 10.1371/journal.pmed.1003513 (PMC7817029; doi:10.1371/journal.pmed.1003513)
Supplement: S5 Table — NPX, normalized protein expression; PEA, proximity extension assay; STABILITY, STabilization of Atherosclerotic plaque By Initiation of darapLadIb TherapY. (PDF) [file pmed.1003513.s007.pdf]

	Cystatin C	GDF-15	IL-6	NT-Pro-BNP	Troponin-T
Cystatin C (lab log2)					
GDF-15 (lab log2)	0.52*				
IL-6 (lab log2)	0.29*	0.29*			
NT-Pro-BNP (lab log2)	0.44*	0.32*	0.25*		
Troponin-T (lab log2)	0.47*	0.43*	0.25*	0.47*	
HSCRP (lab log2)	0.19*	0.14*	0.54*	0.16*	0.14*
GFR (lab log2)	-0.70*	-0.42*	-0.16*	-0.31*	-0.35*
CKD-EPI (lab log2)	-0.73*	-0.46*	-0.20*	-0.35*	-0.39*
HB (lab log2)	-0.23*	-0.31*	-0.11*	-0.23*	-0.13*
BNP	0.32*	0.25*	0.17*	0.78*	0.38*
CCL23	0.35*	0.14*	0.22*	0.25*	0.22*
CCL25	0.32*	0.27*	0.11*	0.22*	0.23*
CCL3	0.31*	0.24*	0.25*	0.15*	0.18*
CD40	0.50*	0.34*	0.23*	0.27*	0.29*
CD5	0.42*	0.30*	0.20*	0.19*	0.17*
CHI3L1	0.30*	0.32*	0.29*	0.22*	0.23*
CSF-1	0.42*	0.30*	0.40*	0.24*	0.26*
CST5	0.40*	0.29*	0.14*	0.22*	0.22*
CSTB	0.50*	0.35*	0.29*	0.26*	0.28*
CX3CL1	0.46*	0.31*	0.14*	0.26*	0.24*
CXCL9	0.46*	0.32*	0.23*	0.30*	0.26*
FABP4	0.45*	0.35*	0.25*	0.23*	0.25*
FAS	0.34*	0.26*	0.15*	0.12*	0.21*
FGF-23	0.33*	0.29*	0.20*	0.18*	0.24*
GDF-15	0.47*	0.86*	0.33*	0.32*	0.42*
HGF	0.23*	0.25*	0.35*	0.15*	0.20*
hK11	0.41*	0.28*	0.14*	0.22*	0.24*
IL-10RB	0.49*	0.35*	0.24*	0.20*	0.24*
IL-12B	0.37*	0.22*	0.18*	0.20*	0.20*
IL-15RA	0.43*	0.26*	0.16*	0.21*	0.21*
IL-6	0.27*	0.27*	0.86*	0.23*	0.21*
KLK6	0.41*	0.24*	0.08*	0.17*	0.15*
LIF-R	0.23*	0.31*	0.15*	0.26*	0.25*
MB	0.39*	0.22*	0.14*	0.12*	0.38*
MMP-12	0.35*	0.36*	0.28*	0.25*	0.24*
NT-pro-BNP	0.31*	0.22*	0.12*	0.75*	0.34*
OPG	0.26*	0.34*	0.27*	0.21*	0.24*
PD-L1	0.42*	0.26*	0.20*	0.19*	0.24*
PlGF	0.55*	0.40*	0.31*	0.29*	0.37*
RETN	0.39*	0.27*	0.24*	0.22*	0.24*
SPON1	0.34*	0.36*	0.22*	0.30*	0.29*
TF	0.39*	0.28*	0.10*	0.24*	0.23*
TGF-alpha	0.38*	0.31*	0.26*	0.23*	0.22*
TIM	0.30*	0.43*	0.20*	0.20*	0.27*
TM	0.38*	0.21*	0.12*	0.11*	0.20*
TNF-R1	0.66*	0.48*	0.36*	0.30*	0.40*
TNF-R2	0.65*	0.48*	0.36*	0.30*	0.35*
TNFRSF9	0.58*	0.36*	0.28*	0.32*	0.30*

	Cystatin C	GDF-15	IL-6	NT-Pro-BNP	Troponin-T
TRAIL-R2	0.53*	0.50*	0.33*	0.32*	0.35*
U-PAR	0.43*	0.35*	0.31*	0.29*	0.27*
VEGF-A	0.32*	0.26*	0.28*	0.16*	0.20*

*p<0.001
