# Supplementary material for: Plasma proteins associated with cardiovascular death in patients with chronic coronary heart disease: A retrospective study
Source: PLoS Med. 2021 Jan 13;18(1):e1003513. doi: 10.1371/journal.pmed.1003513 (PMC7817029; doi:10.1371/journal.pmed.1003513)
Supplement: S6 Table — CV, cardiovascular; LURIC, Ludwigshafen Risk and Cardiovascular Health; NPX, normalized protein expression; PEA, proximity extension assay; STABILITY, STabilization of Atherosclerotic plaque By Initiation of darapLadIb TherapY. (PDF) [file pmed.1003513.s008.pdf]

A

	No event (N=2,788)	Cardiovascular death (N=605)	P-value*
4E-BP1	7.569/8.157/8.889	7.644/8.316/9.026	0.0235
ADA	4.447/4.703/4.995	4.424/4.697/4.980	0.657
AGRP	5.675/5.910/6.169	5.759/6.072/6.365	< 0.0001
AM	9.166/ 9.513/ 9.830	9.393/ 9.775/10.134	< 0.0001
ARTN	0.3192/0.31920.3192	0.3192/0.319/0.3192	0.0668
AXIN1	0.9893/0.989/3.0644	0.9893/0.989/2.9406	0.5226
Beta-NGF	2.516/2.653/2.801	2.602/2.748/2.908	< 0.0001
BNP	1.596/3.341/4.336	3.515/4.666/6.295	< 0.0001
CA-125	6.316/6.771/7.252	6.453/6.955/7.526	< 0.0001
CASP-8	2.625/3.026/3.717	2.704/3.107/3.710	0.0505
CCL11	7.853/8.117/8.405	7.953/8.294/8.615	< 0.0001
CCL19	8.772/9.231/9.853	8.942/9.388/9.995	< 0.0001
CCL20	9.097/ 9.636/10.321	9.330/ 9.904/10.671	< 0.0001
CCL23	9.262/ 9.558/ 9.875	9.378/ 9.747/10.089	< 0.0001
CCL25	6.277/6.701/7.088	6.586/6.953/7.431	< 0.0001
CCL28	1.183/1.373/1.600	1.308/1.522/1.764	< 0.0001
CCL3	3.649/3.931/4.234	3.824/4.118/4.418	< 0.0001
CCL4	8.001/8.371/8.781	8.064/8.390/8.765	0.3342
CD244	5.891/6.104/6.322	5.933/6.146/6.381	< 0.0001
CD40	9.97/10.22/10.51	10.13/10.43/10.79	< 0.0001
CD40-L	7.293/8.082/9.423	7.258/8.070/9.335	0.5915
CD5	3.711/3.941/4.191	3.793/4.057/4.323	< 0.0001
CD6	3.220/3.522/3.813	3.226/3.525/3.824	0.7
CDCP1	3.374/3.744/4.166	3.575/3.941/4.435	< 0.0001
CHI3L1	7.799/8.417/9.212	8.337/8.978/9.881	< 0.0001
CSF-1	10.96/11.12/11.27	11.04/11.22/11.41	< 0.0001
CST5	6.633/6.972/7.319	6.818/7.135/7.567	< 0.0001
CSTB	6.168/6.494/6.847	6.354/6.702/7.124	< 0.0001
CTSD	8.458/8.757/9.098	8.551/8.883/9.206	< 0.0001
CTSL1	7.516/7.708/7.917	7.608/7.824/8.073	< 0.0001
CX3CL1	6.937/7.206/7.489	7.070/7.365/7.676	< 0.0001
CXCL1	7.116/8.231/9.149	7.140/8.107/9.154	0.5917
CXCL10	9.053/ 9.506/10.040	9.198/ 9.693/10.212	< 0.0001
CXCL11	6.008/6.538/7.168	6.156/6.737/7.380	< 0.0001
CXCL16	5.433/5.610/5.786	5.499/5.684/5.886	< 0.0001
CXCL5	8.582/10.03/11.363	8.647/10.03/11.388	0.7776
CXCL6	8.219/8.883/9.611	8.252/8.873/9.686	0.5572
CXCL9	7.664/8.157/8.711	7.987/8.473/9.075	< 0.0001
Dkk-1	6.513/7.020/7.604	6.490/7.024/7.650	0.8243
DNER	7.038/7.216/7.403	6.960/7.160/7.351	< 0.0001
ECP	6.464/7.134/7.917	6.712/7.337/8.106	< 0.0001

	No event (N=2,788)	Cardiovascular death (N=605)	P-value*
EGF	4.927/6.094/7.584	4.920/6.076/7.459	0.8691
EN-RAGE	2.933/3.289/3.823	3.146/3.530/4.149	< 0.0001
ESM-1	4.950/5.218/5.488	5.000/5.307/5.694	< 0.0001
FABP4	4.757/5.205/5.624	4.984/5.437/5.942	< 0.0001
FAS	10.17/10.38/10.58	10.25/10.46/10.67	< 0.0001
FGF-19	6.960/7.558/8.237	7.066/7.701/8.378	0.0235
FGF-21	4.230/4.972/5.713	4.457/5.207/6.083	< 0.0001
FGF-23	3.414/3.746/4.099	3.594/3.942/4.569	< 0.0001
FGF-5	0.7824/1.648/1.8053	0.7824/1.723/1.9054	< 0.0001
Flt3L	8.884/9.153/9.428	8.926/9.186/9.486	0.0272
FS	7.612/7.891/8.167	7.700/7.986/8.328	< 0.0001
GAL	7.366/7.827/8.329	7.301/7.828/8.325	0.5137
Gal-3	7.398/7.671/7.943	7.518/7.759/8.041	< 0.0001
GDF-15	10.62/11.03/11.45	11.01/11.46/11.98	< 0.0001
GDNF	2.088/2.299/2.514	2.161/2.381/2.658	< 0.0001
GH	7.918/ 9.456/10.972	8.741/10.229/11.781	< 0.0001
HB-EGF	7.830/8.105/8.471	7.897/8.172/8.542	0.0059
HGF	7.950/8.198/8.470	8.121/8.397/8.738	< 0.0001
hK11	6.666/6.934/7.202	6.812/7.084/7.425	< 0.0001
HSP-27	4.410/5.401/7.331	4.558/5.504/7.072	0.2927
IFN-gamma	0.6854/0.685/0.6854	0.6854/0.685/0.6854	0.2897
IL-1-alpha	0.7091/0.709/0.7091	0.7091/0.709/0.7091	0.0572
IL-10RA	0.6456/0.646/0.6456	0.6456/0.646/0.646	0.9947
IL-10RB	7.404/7.620/7.819	7.480/7.700/7.949	< 0.0001
IL-12B	4.229/4.638/5.106	4.350/4.735/5.227	< 0.0001
IL-15RA	1.312/1.469/1.624	1.394/1.562/1.761	< 0.0001
IL-16	5.785/6.093/6.395	5.850/6.148/6.495	6e-04
IL-17A	0.500/0.500/1.162	0.500/0.500/1.249	0.0019
IL-17C	0.9001/0.900/1.9323	0.9001/0.900/2.0091	0.0106
IL-18	11.65/12.00/12.37	11.67/12.02/12.42	0.35
IL-18R1	7.319/7.594/7.876	7.390/7.656/8.022	< 0.0001
IL-1ra	3.063/3.063/6.258	3.063/3.063/6.337	0.0502
IL-20	0.6101/0.610/0.6101	0.6101/0.610/0.6101	0.4744
IL-20RA	0.653/0.653/0.653	0.653/0.653/0.653	0.1865
IL-24	0.8909/0.891/0.8909	0.8909/0.891/0.8909	0.5165
IL-2RB	0.6362/0.636/0.6362	0.6362/0.636/0.6362	0.2683
IL-4	1.738/1.738/1.738	1.738/1.738/1.738	0.9792
IL-5	0.9825/0.983/0.9825	0.9825/0.983/0.9825	0.0923
IL-6	4.358/4.857/5.384	4.693/5.289/5.920	< 0.0001
IL-6RA	8.679/8.980/9.229	8.741/9.032/9.280	0.0025
IL-8	6.324/6.712/7.133	6.508/6.910/7.398	< 0.0001
IL10	3.763/4.034/4.328	3.870/4.168/4.509	< 0.0001
IL13	0.8042/0.804/0.8042	0.8042/0.804/0.8042	0.338

	No event (N=2,788)	Cardiovascular death (N=605)	P-value*
IL27-A	5.861/6.116/6.352	5.965/6.255/6.512	< 0.0001
IL33	0.8744/0.874/0.8744	0.8744/0.874/0.8744	0.8978
IL7	2.510/3.093/3.755	2.516/3.061/3.739	0.7383
ITGB1BP2	2.48/2.48/2.48	2.48/2.48/2.48	0.0733
KLK6	7.202/7.483/7.753	7.250/7.568/7.913	< 0.0001
LAP-TGF-beta-1	6.417/6.709/7.090	6.500/6.840/7.241	< 0.0001
LEP	4.836/5.537/6.189	4.756/5.562/6.191	0.8736
LIF	0.4085/0.408/0.4085	0.4085/0.408/0.4085	0.9027
LIF-R	3.294/3.447/3.606	3.371/3.556/3.773	< 0.0001
LOX-1	6.971/7.348/7.784	7.174/7.588/7.994	< 0.0001
mAmP	2.052/4.465/5.342	2.052/4.277/5.200	0.0175
MB	8.453/8.853/9.262	8.512/8.980/9.439	< 0.0001
MCP-1	9.267/9.510/9.790	9.370/9.621/9.948	< 0.0001
MCP-2	8.641/9.028/9.422	8.689/9.112/9.542	0.0011
MCP-3	1.965/2.220/2.557	2.099/2.341/2.685	< 0.0001
MCP-4	2.092/2.471/2.897	2.169/2.577/3.042	0.0011
MMP-1	4.819/5.422/6.130	4.878/5.630/6.286	0.002
MMP-10	8.832/9.153/9.531	8.954/9.302/9.712	< 0.0001
MMP-12	9.347/ 9.798/10.282	9.694/10.169/10.704	< 0.0001
MMP-3	-1.021/-0.610/-0.205	-0.882/-0.435/-0.020	< 0.0001
MMP-7	8.120/8.898/9.427	8.161/9.110/9.747	< 0.0001
MPO	7.334/7.512/7.690	7.403/7.579/7.774	< 0.0001
NEMO	4.680/5.183/6.133	4.732/5.262/6.140	0.2098
NRTN	0.7392/0.739/0.7392	0.7392/0.739/0.7392	0.1428
NT-3	2.046/2.276/2.530	2.089/2.326/2.633	< 0.0001
NT-pro-BNP	6.459/7.376/7.917	7.592/7.986/8.239	< 0.0001
OPG	10.88/11.11/11.37	11.05/11.31/11.60	< 0.0001
OSM	1.852/2.334/2.898	2.067/2.547/3.096	< 0.0001
PAPPA	2.848/3.195/3.518	2.872/3.235/3.608	0.0464
PAR-1	8.128/8.488/8.864	8.230/8.636/9.027	< 0.0001
PD-L1	4.562/4.786/5.027	4.708/4.965/5.234	< 0.0001
PDGF-Subunit-B	7.084/8.130/9.092	6.971/8.090/9.167	0.5262
PECAM-1	7.625/7.873/8.122	7.694/7.975/8.261	< 0.0001
PlGF	8.004/8.207/8.419	8.133/8.377/8.652	< 0.0001
PRL	5.048/5.471/5.916	5.127/5.565/6.073	0.0009
PSGL-1	1.975/2.089/2.203	1.975/2.088/2.213	0.936
PTX3	2.197/2.502/2.836	2.338/2.675/3.041	< 0.0001
RAGE	6.908/7.231/7.520	7.055/7.399/7.749	< 0.0001
REN	8.191/8.869/9.538	8.438/9.220/9.946	< 0.0001
RETN	7.518/7.882/8.252	7.686/8.063/8.494	< 0.0001
SCF	8.614/8.905/9.158	8.540/8.879/9.149	0.1055
SELE	6.646/7.084/7.497	6.655/7.080/7.522	0.763
SIRT2	3.886/4.689/6.308	3.888/4.665/6.119	0.5047

	No event (N=2,788)	Cardiovascular death (N=605)	P-value*
SLAMF1	3.234/3.521/3.848	3.402/3.706/4.097	< 0.0001
SPON1	7.009/7.191/7.370	7.180/7.361/7.594	< 0.0001
SRC	5.146/6.610/7.862	5.139/6.505/7.892	0.8855
ST1A1	0.9613/1.9521/3.4674	1.0176/1.950/3.2354	0.8167
ST2	5.743/6.093/6.453	5.929/6.271/6.671	< 1e-04
STAMPB	3.261/3.732/4.749	3.281/3.725/4.655	0.8232
t-PA	11.36/11.68/11.93	11.29/11.67/11.97	0.8656
TF	5.207/5.419/5.619	5.288/5.490/5.771	< 0.0001
TGF-alpha	1.289/1.482/1.696	1.457/1.659/1.928	< 0.0001
TIE2	8.358/8.528/8.706	8.389/8.573/8.792	< 0.0001
TIM	7.714/8.255/8.842	8.108/8.618/9.367	< 0.0001
TM	11.61/11.80/12.02	11.62/11.83/12.08	0.015
TNF	0.6806/0.680/0.6806	0.6806/0.680/0.6806	0.0601
TNF-R1	12.55/12.79/13.04	12.70/13.02/13.29	< 0.0001
TNF-R2	6.596/6.898/7.237	6.792/7.146/7.549	< 0.0001
TNFB	3.126/3.370/3.606	3.123/3.397/3.668	0.1046
TNFRSF9	6.327/6.626/6.954	6.489/6.855/7.238	< 0.0001
TNFSF14	3.841/4.157/4.535	3.919/4.206/4.598	0.0068
TRAIL	10.18/10.38/10.57	10.15/10.38/10.58	0.3467
TRAIL-R2	2.419/2.640/2.895	2.641/2.930/3.247	< 0.0001
TRANCE	5.006/5.407/5.796	4.980/5.402/5.835	0.9816
TSLP	0.9694/0.969/0.9694	0.9694/0.969/0.9694	0.7288
TWEAK	7.870/8.075/8.272	7.817/8.053/8.232	0.0044
U-PAR	11.02/11.22/11.42	11.20/11.42/11.63	< 0.0001
uPA	9.662/ 9.868/10.071	9.691/ 9.933/10.164	< 0.0001
VEGF-A	9.89/10.118/10.395	10.04/10.285/10.604	< 0.0001
VEGF-D	7.673/7.953/8.225	7.888/8.196/8.589	< 0.0001
4E-BP1	7.569/8.157/8.889	7.644/8.316/9.026	0.0235

* Wilcoxon test

B

	No event (N=1042)	Cardiovascular death (N=245)	P-value*
AGRP	2.118/2.374/2.624	2.200/2.489/2.890	<0.0001
AM	4.624/5.021/5.450	4.981/5.439/5.888	<0.0001
Beta-NGF	-0.34786/-0.34786/-0.20664	-0.34786/-0.18296/ 0.07478	<0.0001
BNP	1.236/1.236/2.487	1.236/1.236/3.840	<0.0001
CA-125	-0.20331/-0.20331/-0.01838	-0.20331/-0.20331/ 0.50593	<0.0001
CASP-8	-0.08268/ 0.26880/ 0.66308	-0.01712/ 0.30383/ 0.66027	0.3792
CCL20	4.436/4.967/5.616	4.655/5.125/6.000	7e-04
CCL3	-0.16188/ 0.13229/ 0.47259	0.05389/ 0.42494/ 0.79362	<0.0001
CCL4	4.462/4.794/5.169	4.626/4.950/5.279	<0.0001
CD40	6.494/6.745/6.994	6.651/6.927/7.320	<0.0001
CD40-L	0.4024/0.4024/0.4024	0.4024/0.4024/0.4024	0.026
CHI3L1	3.351/3.901/4.517	3.844/4.340/5.166	<0.0001
CSF-1	6.982/7.143/7.337	7.100/7.291/7.510	<0.0001
CSTB	1.978/2.271/2.604	2.179/2.512/2.965	<0.0001
CTSD	6.241/6.529/6.850	6.350/6.658/6.965	3e-04
CTSL1	4.186/4.514/4.847	4.404/4.738/5.181	<0.0001
CX3CL1	3.480/3.808/4.129	3.531/3.917/4.299	0.0021
CXCL1	5.860/6.208/6.647	5.911/6.326/6.747	0.0952
CXCL16	2.539/2.732/2.910	2.634/2.816/3.062	<0.0001
CXCL6	5.655/6.016/6.341	5.782/6.103/6.489	0.0048
Dkk-1	1.683/2.017/2.294	1.666/2.036/2.371	0.2398
ECP	1.575/2.228/2.889	1.837/2.443/3.170	0.001
EGF	3.622/4.212/4.817	3.460/4.061/4.697	0.0128
EN-RAGE	0.9416/1.5409/2.1988	1.1439/1.7955/2.4324	0.0011
ESM-1	1.293/1.667/2.140	1.554/1.987/2.853	<0.0001
FABP4	1.660/2.187/2.768	2.048/2.540/3.290	<0.0001
FAS	6.519/6.723/6.938	6.600/6.817/7.021	3e-04
FGF-23	0.9199/1.2430/1.6646	1.1120/1.5466/2.1735	<0.0001
FS	0.1396/0.1396/0.1396	0.1396/0.1396/0.1396	0.0075
GAL	4.410/4.903/5.470	4.341/4.913/5.471	0.9527
Gal-3	3.353/3.607/3.851	3.383/3.633/3.896	0.1687
GDF-15	6.608/7.020/7.444	7.125/7.522/8.013	<0.0001
GH	3.471/4.871/6.429	4.535/5.572/7.231	<0.0001
HB-EGF	4.382/4.665/4.922	4.539/4.847/5.088	<0.0001
HGF	5.051/5.341/5.845	5.312/5.726/7.154	<0.0001
hK11	3.898/4.170/4.434	4.048/4.338/4.690	<0.0001
HSP-27	0.3913/1.2593/1.8943	0.8283/1.3231/1.9720	0.2894
IL-16	2.582/2.894/3.179	2.550/2.983/3.410	0.0139
IL-18	5.203/5.578/5.934	5.209/5.611/5.977	0.4037
IL-1ra	2.603/3.050/3.599	2.749/3.202/3.794	0.0022
IL-6	2.359/2.905/3.669	2.816/3.444/4.452	<0.0001
IL-6RA	5.293/5.566/5.830	5.330/5.644/5.862	0.0334

	No event (N=1042)	Cardiovascular death (N=245)	P-value*
IL-8	4.501/4.793/5.094	4.705/5.075/5.511	<0.0001
IL27-A	0.9132/1.1707/1.4312	1.0931/1.3886/1.7137	<0.0001
KLK6	3.028/3.286/3.578	3.038/3.294/3.573	0.764
LEP	4.019/4.732/5.382	3.793/4.740/5.410	0.9772
LOX-1	3.975/4.365/4.802	4.099/4.471/4.900	0.0091
mAmP	0.8135/0.8135/2.2701	0.8135/0.8135/2.2395	0.9773
MB	4.254/4.549/4.927	4.327/4.726/5.179	<0.0001
MCP-1	8.479/8.714/8.979	8.601/8.836/9.180	<0.0001
MMP-1	-2.670/-2.670/-1.539	-2.670/-2.670/-1.288	0.0011
MMP-10	6.998/7.360/7.743	7.106/7.411/7.877	0.0188
MMP-12	6.176/6.712/7.255	6.505/7.045/7.606	<0.0001
MMP-3	5.413/5.828/6.219	5.520/5.952/6.387	0.0043
MMP-7	6.466/6.777/7.156	6.671/7.045/7.454	<0.0001
MPO	3.472/3.667/3.852	3.547/3.722/3.911	0.0022
NEMO	1.356/1.803/2.250	1.360/1.832/2.343	0.5587
NT-pro-BNP	1.004/3.302/4.588	3.539/4.900/6.198	<0.0001
OPG	8.324/8.591/8.868	8.611/8.847/9.154	<0.0001
PAPPA	0.1811/0.1811/0.1811	0.1811/0.1811/0.1811	0.5697
PAR-1	4.934/5.260/5.570	5.080/5.420/5.731	<0.0001
PDGF-Subunit-B	1.634/2.223/2.796	1.621/2.190/2.725	0.4303
PECAM-1	3.428/3.648/3.875	3.447/3.667/3.893	0.3493
PlGF	5.508/5.728/5.954	5.674/5.898/6.211	<0.0001
PRL	1.349/1.768/2.194	1.410/1.793/2.251	0.436
PSGL-1	-2.968/-2.968/-2.968	-2.968/-2.968/-2.968	0.9339
PTX3	0.1673/0.1673/0.1673	0.1673/0.1673/0.1673	0.0059
RAGE	2.508/2.810/3.123	2.589/2.917/3.308	9e-04
REN	5.361/6.034/6.689	5.835/6.567/7.379	<0.0001
RETN	7.998/8.306/8.629	8.145/8.425/8.913	<0.0001
SCF	6.916/7.301/7.622	6.841/7.271/7.617	0.1834
SELE	2.726/3.173/3.600	2.632/3.160/3.531	0.148
SIRT2	0.9941/1.4454/1.9649	1.1234/1.5379/2.0426	0.033
SPON1	0.9935/1.2406/1.4811	1.2133/1.4605/1.7363	<0.0001
SRC	4.137/4.849/5.569	3.971/4.710/5.478	0.0917
ST2	2.122/2.477/2.828	2.318/2.744/3.241	<0.0001
t-PA	4.219/4.547/4.867	4.344/4.687/5.058	<0.0001
TF	2.996/3.187/3.384	3.051/3.278/3.532	<0.0001
TIE2	4.175/4.338/4.534	4.190/4.371/4.596	0.1539
TIM	5.456/5.951/6.556	6.026/6.531/7.107	<0.0001
TM	8.813/9.021/9.247	8.841/9.077/9.411	0.0048
TNF-R1	10.29/10.53/10.79	10.47/10.75/11.10	<0.0001
TNF-R2	3.285/3.569/3.908	3.443/3.762/4.280	<0.0001
TNFSF14	-0.73033/-0.44963/-0.10803	-0.66026/-0.38024/ 0.05890	0.0041
TRAIL	6.253/6.467/6.681	6.198/6.450/6.687	0.4259
TRAIL-R2	1.088/1.307/1.599	1.297/1.587/2.017	<0.0001

	No event (N=1042)	Cardiovascular death (N=245)	P-value*
TRANCE	2.541/2.965/3.357	2.271/2.763/3.315	3e-04
U-PAR	7.683/7.960/8.221	7.900/8.186/8.475	<0.0001
VEGF-A	7.658/7.864/8.084	7.793/8.054/8.400	<0.0001
VEGF-D	5.656/5.974/6.353	5.801/6.149/6.536	<0.0001

* Wilcoxon test
