# Supplementary figures and images for: Plasma proteins associated with cardiovascular death in patients with chronic coronary heart disease: A retrospective study
Source: PLoS Med. 2021 Jan 13;18(1):e1003513. doi: 10.1371/journal.pmed.1003513 (PMC7817029; doi:10.1371/journal.pmed.1003513)

A

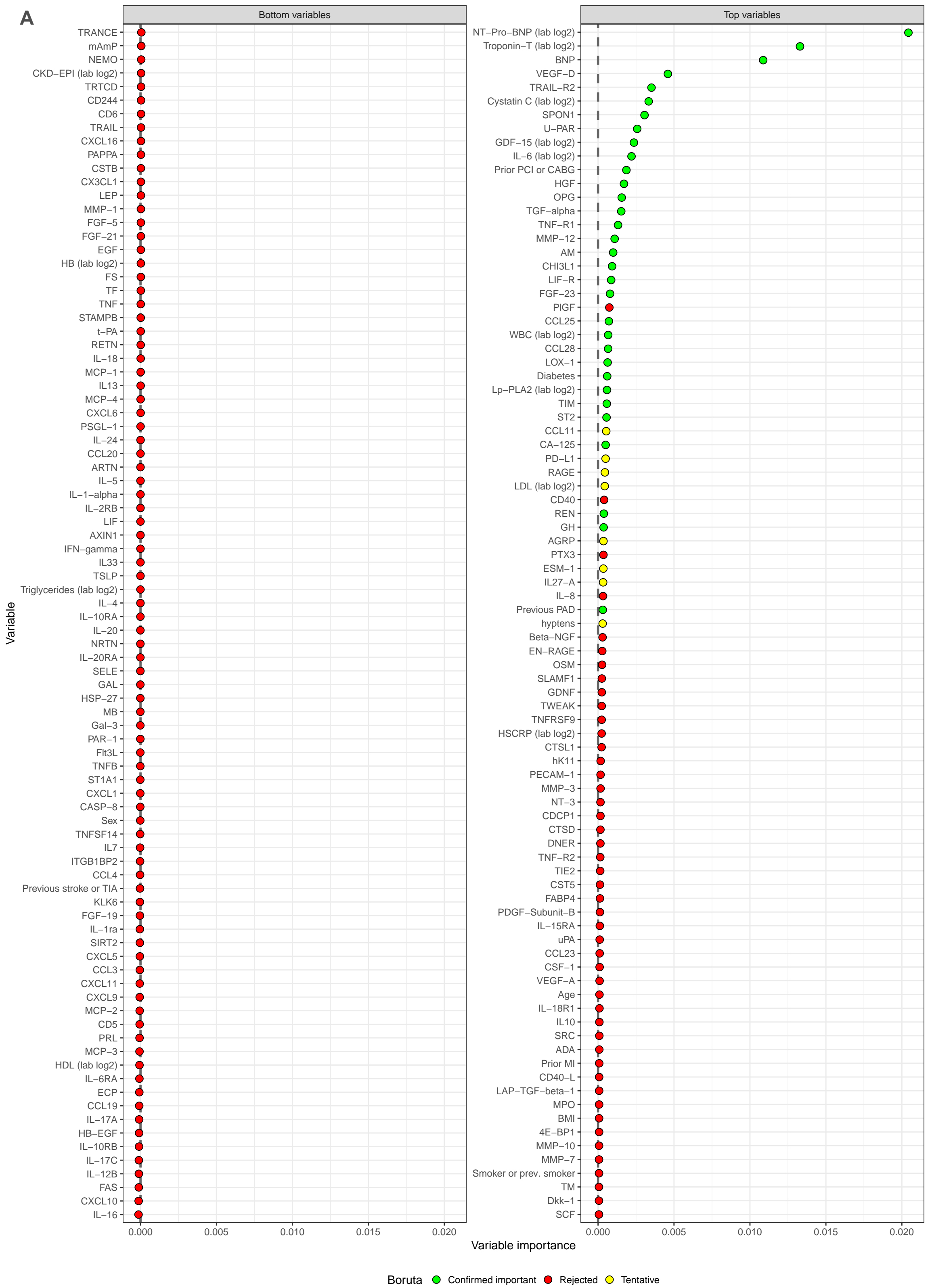

B

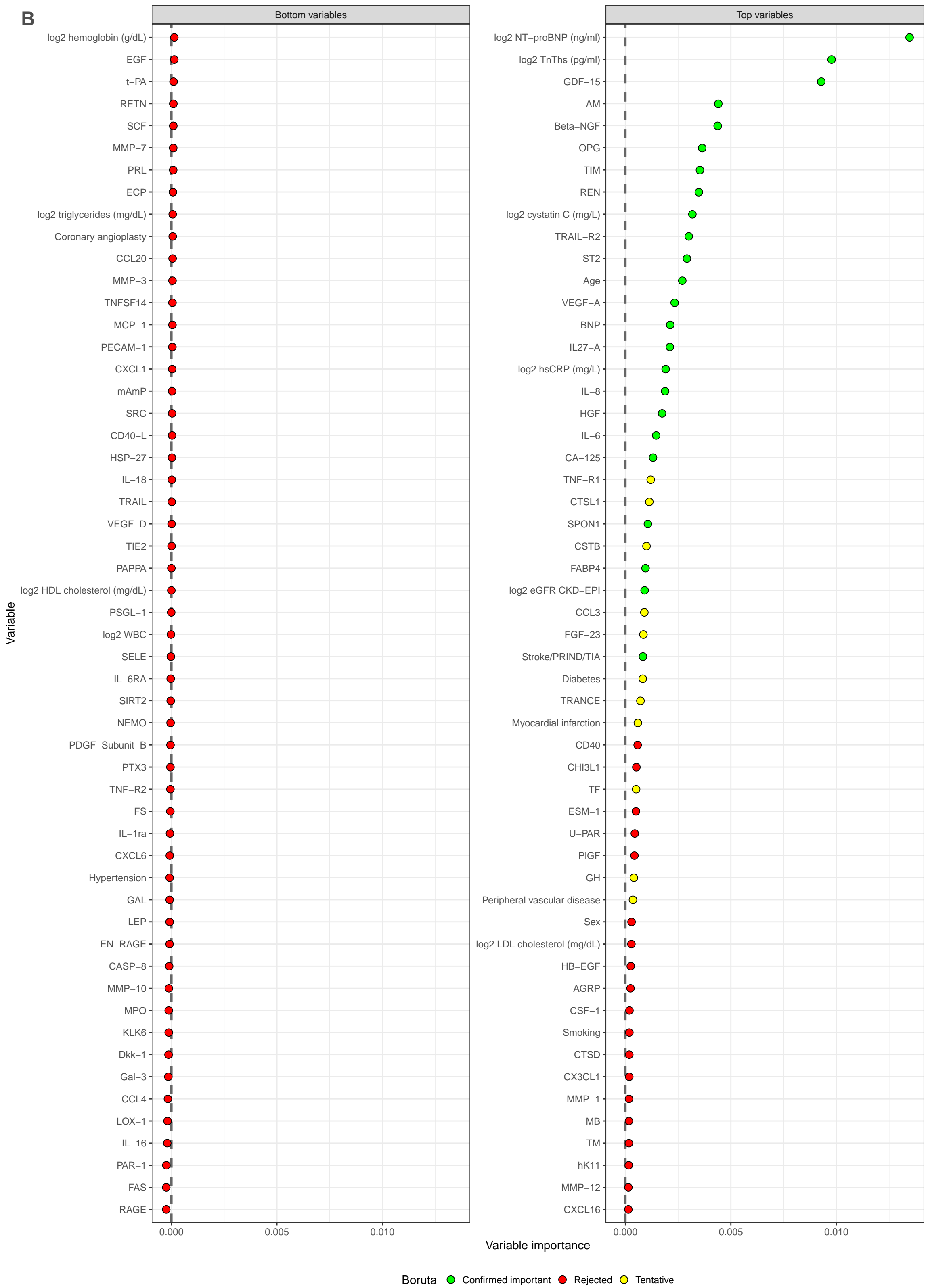

Supplement: S1 Fig — Each estimate of variable importance is colored by the Boruta analysis result. CV, cardiovascular; LURIC, Ludwigshafen Risk and Cardiovascular Health; PEA, proximity extension assay; RF, Random Survival Forest; STABILITY, STabilization of Atherosclerotic plaque By Initiation of darapLadIb TherapY. (PDF) [file pmed.1003513.s009.pdf]
