# Supplementary material for: Plasma proteins associated with cardiovascular death in patients with chronic coronary heart disease: A retrospective study
Source: PLoS Med. 2021 Jan 13;18(1):e1003513. doi: 10.1371/journal.pmed.1003513 (PMC7817029; doi:10.1371/journal.pmed.1003513)

A

Variable	By SD	N	HR [95% CI]	p−value
NT−pro−BNP	1.21	3393	3.16 [2.57,3.87]	3.21e−28
NT−Pro−BNP (lab log2)	1.74	3393	2.95 [2.65,3.27]	1.19e−88
BNP	1.84	3393	2.37 [2.14,2.62]	3.95e−63
Troponin−T (lab log2)	0.97	3393	2.14 [1.96,2.34]	5.39e−64
VEGF−D	0.53	3393	2.07 [1.80,2.37]	8.49e−26
SPON1	0.31	3393	2.05 [1.83,2.29]	1.87e−36
GDF−15	0.68	3393	1.94 [1.77,2.13]	3.40e−45
GDF−15 (lab log2)	0.79	3393	1.88 [1.72,2.05]	1.48e−44
Cystatin C (lab log2)	0.38	3393	1.80 [1.65,1.96]	8.57e−40
U−PAR	0.35	3393	1.76 [1.59,1.95]	2.19e−27
OPG	0.40	3393	1.75 [1.57,1.94]	7.06e−26
TNF−R1	0.40	3393	1.70 [1.55,1.87]	1.41e−27
MMP−12	0.74	3393	1.66 [1.52,1.81]	2.23e−29
PlGF	0.37	3393	1.66 [1.50,1.82]	1.20e−24
IL−6 (lab log2)	0.96	3393	1.65 [1.53,1.79]	6.69e−35
HGF	0.45	3393	1.63 [1.50,1.78]	1.62e−28
LIF−R	0.28	3393	1.62 [1.46,1.80]	1.04e−19
hK11	0.61	3393	1.59 [1.35,1.87]	2.26e−08
ST2	0.59	3393	1.58 [1.43,1.75]	2.40e−18
TNF−R2	0.53	3393	1.58 [1.44,1.72]	3.99e−24
CHI3L1	1.17	3393	1.56 [1.44,1.68]	2.29e−28
CCL25	0.64	3393	1.55 [1.39,1.73]	4.25e−15
TIM	0.91	3393	1.54 [1.42,1.66]	1.19e−25
CD40	0.46	3393	1.53 [1.41,1.67]	1.97e−23
IL−15RA	0.41	3393	1.52 [1.35,1.70]	1.20e−12
IL−6	0.95	3393	1.48 [1.38,1.59]	6.54e−27
CA−125	0.92	3393	1.48 [1.34,1.63]	3.29e−14
FABP4	0.73	3393	1.47 [1.34,1.61]	3.13e−16
RAGE	0.49	3393	1.47 [1.31,1.65]	9.04e−11
CSTB	0.57	3393	1.46 [1.34,1.59]	3.74e−18
IL27−A	0.40	3393	1.45 [1.32,1.59]	1.46e−14
CX3CL1	0.45	3393	1.44 [1.31,1.59]	2.71e−14
CST5	0.54	3393	1.44 [1.32,1.57]	1.75e−16
CSF−1	0.27	3393	1.44 [1.28,1.61]	1.07e−09
GH	2.00	3393	1.44 [1.31,1.57]	1.52e−15
CCL23	0.51	3393	1.43 [1.28,1.60]	3.56e−10
FGF−23	0.75	3393	1.42 [1.32,1.53]	1.07e−19
TNFRSF9	0.55	3393	1.42 [1.32,1.53]	6.04e−20
WBC (lab log2)	0.39	3393	1.42 [1.30,1.54]	1.65e−15
CXCL9	0.86	3393	1.41 [1.30,1.52]	1.43e−18
SLAMF1	0.54	3393	1.41 [1.30,1.52]	5.20e−17
HSCRP (lab log2)	1.70	3393	1.40 [1.28,1.54]	2.80e−13
CDCP1	0.63	3393	1.40 [1.29,1.52]	2.50e−16
REN	0.99	3393	1.39 [1.26,1.54]	1.25e−10
PTX3	0.55	3393	1.38 [1.26,1.50]	2.53e−13
EN−RAGE	1.06	3393	1.37 [1.27,1.48]	8.79e−16
RETN	0.64	3393	1.37 [1.26,1.49]	1.41e−13
ESM−1	0.44	3393	1.37 [1.23,1.52]	4.14e−09
Beta−NGF	0.68	3393	1.37 [1.25,1.49]	1.75e−12
CCL11	0.52	3393	1.36 [1.20,1.53]	6.35e−07
VEGF−A	0.49	3393	1.35 [1.26,1.46]	2.30e−15
AM	1.08	3393	1.35 [1.04,1.77]	2.65e−02
FS	0.45	3393	1.35 [1.23,1.47]	1.90e−10
TGF−alpha	0.44	3393	1.34 [1.26,1.43]	9.33e−21
CXCL16	0.32	3393	1.33 [1.20,1.48]	1.64e−07
TRAIL−R2	0.49	3393	1.33 [1.23,1.44]	5.21e−12
CTSL1	0.39	3393	1.32 [1.23,1.42]	8.39e−14
TF	0.34	3393	1.32 [1.20,1.45]	9.85e−09
IL−10RB	0.34	3393	1.31 [1.18,1.45]	1.60e−07
Gal−3	0.42	3393	1.30 [1.19,1.44]	5.60e−08
GDNF	0.47	3393	1.30 [1.18,1.44]	4.61e−07
PD−L1	0.49	3393	1.30 [1.21,1.40]	2.50e−12
MMP−3	0.68	3393	1.30 [1.19,1.41]	2.32e−09
Lp−PLA2 (lab log2)	0.43	3393	1.29 [1.16,1.43]	1.36e−06
CD5	0.41	3393	1.28 [1.18,1.39]	1.46e−09
OSM	0.89	3393	1.28 [1.18,1.39]	6.07e−09
AGRP	0.52	3393	1.27 [1.17,1.39]	6.43e−08
CCL28	0.44	3393	1.27 [1.18,1.36]	1.01e−10
IL−18R1	0.44	3393	1.27 [1.15,1.39]	8.80e−07
CCL20	1.08	3393	1.26 [1.18,1.36]	1.77e−10
MCP−3	0.79	3393	1.26 [1.17,1.36]	2.57e−09
LOX−1	0.76	3393	1.26 [1.17,1.35]	1.52e−10
MMP−10	0.59	3393	1.25 [1.15,1.37]	2.75e−07
PECAM−1	0.41	3393	1.25 [1.14,1.37]	8.46e−07
IL−8	0.92	3393	1.24 [1.16,1.33]	6.54e−11
MPO	0.35	3393	1.24 [1.14,1.34]	1.01e−07
FGF−5	0.53	3393	1.24 [1.13,1.35]	2.39e−06
CTSD	0.47	3393	1.24 [1.13,1.35]	2.21e−06
KLK6	0.46	3393	1.23 [1.12,1.35]	1.87e−05
FGF−21	1.22	3393	1.23 [1.12,1.34]	3.35e−06
FAS	0.37	3393	1.22 [1.13,1.32]	3.84e−07
uPA	0.38	3393	1.22 [1.12,1.33]	3.28e−06
TIE2	0.30	3393	1.22 [1.10,1.35]	9.97e−05
CXCL10	0.89	3393	1.20 [1.10,1.31]	2.64e−05
CCL3	0.67	3393	1.20 [1.13,1.27]	1.32e−09
MCP−1	0.50	3393	1.19 [1.10,1.29]	1.26e−05
CXCL11	0.97	3393	1.19 [1.09,1.29]	4.70e−05
LAP−TGF−beta−1	0.59	3393	1.18 [1.09,1.28]	3.50e−05
IL−12B	0.70	3393	1.17 [1.07,1.29]	7.08e−04
MB	0.65	3393	1.17 [1.07,1.28]	7.11e−04
CD244	0.36	3393	1.17 [1.08,1.27]	1.87e−04
IL10	0.77	3393	1.16 [1.09,1.25]	2.26e−05
PRL	0.76	3393	1.16 [1.06,1.26]	1.34e−03
ECP	1.21	3393	1.16 [1.06,1.26]	9.24e−04
PAR−1	0.78	3393	1.15 [1.02,1.29]	1.98e−02
IL−6RA	0.39	3393	1.15 [1.05,1.26]	3.49e−03
TM	0.34	3393	1.14 [1.03,1.26]	1.51e−02
IL−17C	0.76	3393	1.13 [1.05,1.23]	2.12e−03
NT−3	0.49	3393	1.13 [1.04,1.23]	3.19e−03
MCP−2	0.72	3393	1.13 [1.03,1.23]	6.71e−03
MMP−1	1.05	3393	1.12 [1.03,1.22]	7.68e−03
MCP−4	0.69	3393	1.12 [1.03,1.21]	6.54e−03
CCL19	0.98	3393	1.12 [1.03,1.22]	1.10e−02
MMP−7	1.81	3393	1.11 [0.99,1.24]	6.87e−02
TNFSF14	0.68	3393	1.11 [1.02,1.20]	1.25e−02
IL−16	0.57	3393	1.11 [1.00,1.22]	4.19e−02
LDL (lab log2)	0.55	3393	1.10 [0.99,1.22]	6.46e−02
IL−17A	0.58	3393	1.09 [1.02,1.18]	1.30e−02
IL−1ra	1.72	3393	1.09 [1.00,1.19]	4.15e−02
FGF−19	0.96	3393	1.08 [0.99,1.18]	6.90e−02
Flt3L	0.44	3393	1.08 [0.98,1.19]	1.42e−01
IL−1−alpha	0.33	3393	1.07 [1.00,1.16]	5.89e−02
PAPPA	0.60	3393	1.07 [0.97,1.18]	1.71e−01
CASP−8	1.07	3393	1.07 [0.98,1.16]	1.33e−01
4E−BP1	1.23	3393	1.05 [0.96,1.16]	2.66e−01
TNF	0.76	3393	1.05 [0.97,1.13]	2.17e−01
IL−2RB	0.29	3393	1.05 [0.97,1.13]	2.08e−01
HB−EGF	0.70	3393	1.05 [0.96,1.14]	2.73e−01
TNFB	0.44	3393	1.04 [0.95,1.14]	3.52e−01
NEMO	1.45	3393	1.04 [0.95,1.13]	3.95e−01
ARTN	0.28	3393	1.03 [0.98,1.10]	2.52e−01
CXCL6	1.07	3393	1.03 [0.94,1.12]	5.65e−01
HSP−27	1.97	3393	1.02 [0.94,1.11]	6.27e−01
IL−20	0.38	3393	1.02 [0.94,1.10]	6.52e−01
PSGL−1	0.20	3393	1.02 [0.92,1.12]	7.52e−01
IL−18	0.60	3393	1.01 [0.93,1.10]	7.86e−01
CD6	0.48	3393	1.01 [0.92,1.10]	8.67e−01
IL−4	0.52	3393	1.01 [0.92,1.10]	8.96e−01
CCL4	0.75	3393	1.01 [0.93,1.09]	8.99e−01
SELE	0.68	3393	1.00 [0.92,1.10]	9.49e−01
EGF	1.92	3393	1.00 [0.92,1.08]	9.35e−01
SRC	1.58	3393	0.99 [0.91,1.08]	8.91e−01
IL−10RA	0.49	3393	0.99 [0.91,1.09]	8.93e−01
ADA	0.49	3393	0.99 [0.90,1.09]	8.56e−01
Dkk−1	0.89	3393	0.98 [0.90,1.08]	7.38e−01
TSLP	0.09	3393	0.98 [0.89,1.08]	7.26e−01
CXCL5	1.88	3393	0.98 [0.90,1.07]	6.89e−01
CD40−L	2.01	3393	0.98 [0.90,1.07]	6.76e−01
ST1A1	1.59	3393	0.98 [0.90,1.07]	6.34e−01
CXCL1	1.36	3393	0.98 [0.90,1.06]	5.94e−01
IL33	0.24	3393	0.98 [0.90,1.06]	5.87e−01
PDGF−Subunit−B	1.53	3393	0.98 [0.89,1.07]	5.81e−01
IL7	0.97	3393	0.97 [0.90,1.06]	5.28e−01
SIRT2	2.02	3393	0.97 [0.89,1.05]	4.49e−01
LIF	0.45	3393	0.97 [0.88,1.06]	4.66e−01
AXIN1	1.48	3393	0.96 [0.88,1.05]	3.73e−01
LEP	1.11	3393	0.96 [0.87,1.06]	4.38e−01
t−PA	0.56	3393	0.96 [0.87,1.05]	3.49e−01
STAMPB	1.25	3393	0.96 [0.88,1.04]	3.15e−01
IL−24	0.49	3393	0.96 [0.87,1.05]	3.59e−01
Triglycerides (lab log2)	0.72	3393	0.95 [0.87,1.04]	3.01e−01
IL13	0.49	3393	0.95 [0.87,1.05]	3.07e−01
GAL	0.74	3393	0.95 [0.87,1.04]	2.95e−01
TRANCE	0.75	3393	0.95 [0.86,1.05]	3.20e−01
IL−5	1.03	3393	0.95 [0.86,1.05]	2.92e−01
HDL (lab log2)	0.37	3393	0.94 [0.86,1.03]	1.81e−01
NRTN	0.39	3393	0.93 [0.83,1.04]	1.97e−01
IFN−gamma	0.16	3393	0.92 [0.80,1.06]	2.64e−01
TRAIL	0.34	3393	0.92 [0.84,1.01]	7.57e−02
ITGB1BP2	1.67	3393	0.92 [0.84,1.01]	6.98e−02
IL−20RA	0.39	3393	0.91 [0.83,1.00]	5.32e−02
SCF	0.48	3393	0.90 [0.83,0.98]	2.01e−02
mAmP	1.68	3393	0.90 [0.82,0.98]	1.56e−02
TWEAK	0.40	3393	0.87 [0.82,0.94]	2.23e−04
DNER	0.32	3393	0.86 [0.80,0.93]	1.15e−04
HB (lab log2)	0.15	3393	0.83 [0.76,0.91]	4.92e−05
GFR (lab log2)	0.38	3393	0.71 [0.65,0.78]	1.69e−13
CKD−EPI (lab log2)	0.39	3393	0.68 [0.63,0.74]	2.01e−19

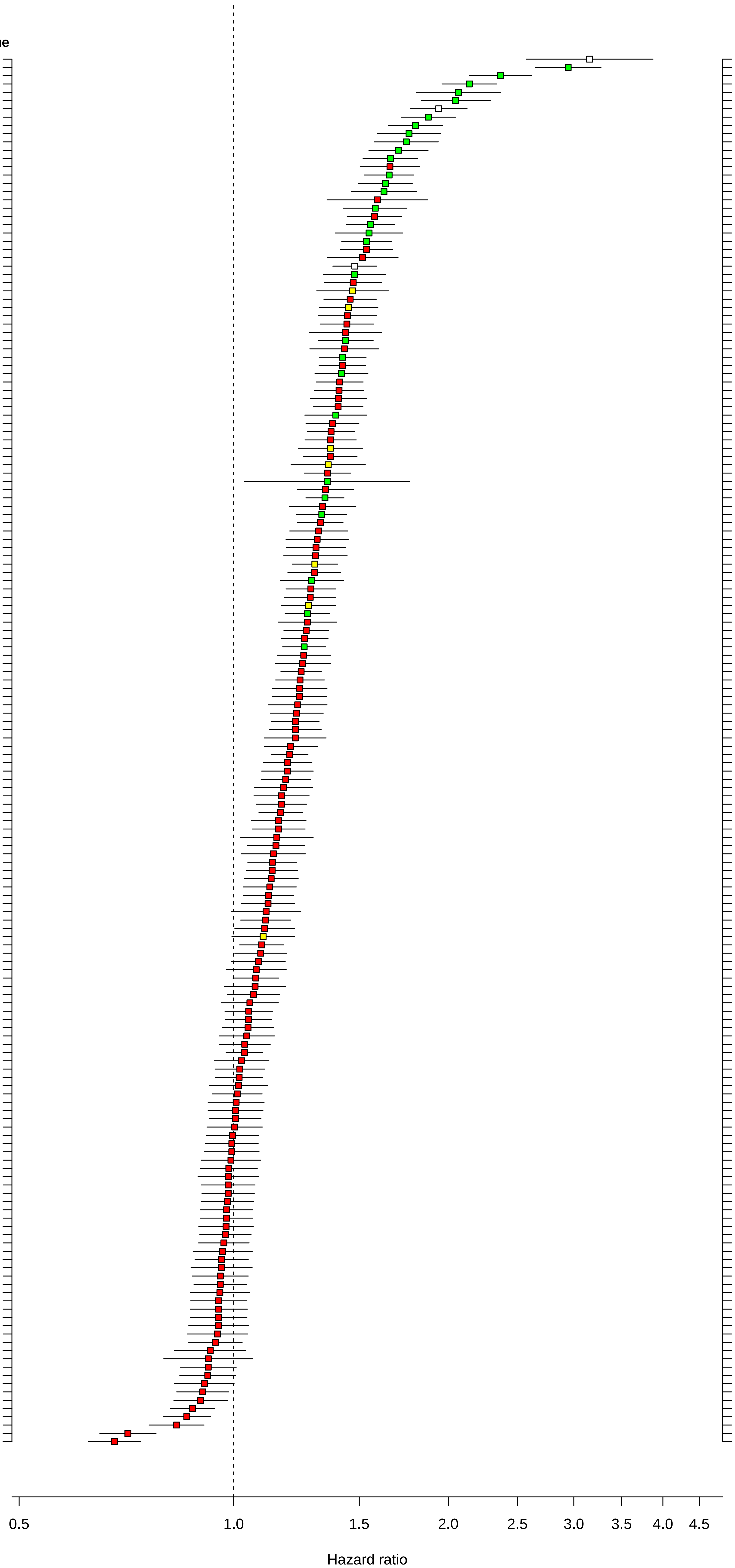

B

Variable	By SD	N	HR [95% CI]	p−value
log2 NT−proBNP (ng/ml)	2.04	1287	2.37 [2.08,2.70]	3.75e−39
NT−pro−BNP	1.90	1287	2.28 [1.99,2.60]	7.11e−33
AM	0.77	1287	2.07 [1.78,2.40]	2.43e−21
log2 TnThs (pg/ml)	1.41	1287	2.07 [1.82,2.34]	1.65e−30
GDF−15	0.74	1287	2.00 [1.80,2.21]	2.74e−39
OPG	0.45	1287	1.81 [1.61,2.03]	7.70e−24
IL27−A	0.45	1287	1.76 [1.56,1.99]	1.43e−19
U−PAR	0.44	1287	1.75 [1.55,1.98]	5.52e−20
TIM	0.93	1287	1.72 [1.54,1.91]	6.96e−23
TNF−R1	0.44	1287	1.70 [1.53,1.90]	1.22e−21
CSF−1	0.29	1287	1.70 [1.50,1.92]	7.17e−17
REN	1.09	1287	1.69 [1.48,1.93]	5.84e−15
ST2	0.62	1287	1.68 [1.50,1.88]	5.16e−19
IL−6	1.24	1287	1.64 [1.48,1.81]	2.37e−21
BNP	1.35	1287	1.63 [1.48,1.80]	2.52e−22
log2 hsCRP (mg/L)	1.76	1287	1.55 [1.37,1.75]	5.92e−12
CHI3L1	1.07	1287	1.54 [1.38,1.72]	1.40e−14
TNF−R2	0.55	1287	1.53 [1.38,1.71]	4.78e−15
FABP4	0.95	1287	1.53 [1.37,1.71]	6.63e−14
MMP−7	0.60	1287	1.52 [1.36,1.70]	4.42e−13
hK11	0.51	1287	1.50 [1.35,1.67]	5.44e−14
ESM−1	0.86	1287	1.49 [1.34,1.66]	4.82e−14
CA−125	0.73	1287	1.48 [1.36,1.61]	4.69e−19
SPON1	0.47	1287	1.47 [1.33,1.63]	1.72e−14
MMP−12	0.85	1287	1.46 [1.29,1.65]	2.22e−09
CSTB	0.57	1287	1.46 [1.33,1.59]	1.32e−16
GH	2.08	1287	1.45 [1.28,1.64]	1.97e−09
log2 cystatin C (mg/L)	0.39	1287	1.45 [1.34,1.56]	5.73e−23
MCP−1	0.41	1287	1.44 [1.29,1.62]	1.77e−10
VEGF−A	0.41	1287	1.43 [1.32,1.55]	7.75e−19
CD40	0.51	1287	1.41 [1.30,1.53]	7.18e−18
HB−EGF	0.64	1287	1.41 [1.22,1.63]	4.43e−06
PAR−1	0.53	1287	1.41 [1.25,1.59]	3.23e−08
CXCL16	0.32	1287	1.41 [1.25,1.58]	1.47e−08
TF	0.35	1287	1.40 [1.25,1.57]	5.29e−09
IL−8	0.71	1287	1.40 [1.30,1.50]	7.03e−21
RETN	0.52	1287	1.39 [1.24,1.57]	3.06e−08
t−PA	0.56	1287	1.39 [1.22,1.59]	1.29e−06
CTSL1	0.63	1287	1.39 [1.26,1.54]	1.53e−10
HGF	1.31	1287	1.38 [1.25,1.52]	5.57e−11
AGRP	0.46	1287	1.38 [1.24,1.53]	1.53e−09
FGF−23	0.94	1287	1.36 [1.26,1.46]	1.60e−16
PlGF	0.46	1287	1.35 [1.24,1.47]	1.93e−12
VEGF−D	0.53	1287	1.34 [1.18,1.52]	3.51e−06
Beta−NGF	0.28	1287	1.33 [1.23,1.42]	1.14e−14
CCL20	1.05	1287	1.32 [1.18,1.47]	6.16e−07
CTSD	0.48	1287	1.31 [1.16,1.48]	1.69e−05
TM	0.36	1287	1.31 [1.15,1.48]	2.13e−05
MB	0.59	1287	1.29 [1.15,1.45]	2.55e−05
IL−1ra	0.82	1287	1.29 [1.14,1.45]	2.87e−05
TNFSF14	0.56	1287	1.28 [1.13,1.45]	8.21e−05
CX3CL1	0.53	1287	1.27 [1.12,1.42]	9.93e−05
RAGE	0.53	1287	1.25 [1.11,1.41]	2.49e−04
CXCL6	0.61	1287	1.25 [1.11,1.41]	1.70e−04
MMP−3	0.76	1287	1.25 [1.10,1.41]	3.60e−04
MMP−1	0.80	1287	1.25 [1.11,1.40]	1.27e−04
TRAIL−R2	0.63	1287	1.25 [1.18,1.31]	4.18e−17
MMP−10	0.63	1287	1.24 [1.10,1.41]	5.27e−04
ECP	1.01	1287	1.24 [1.10,1.41]	6.45e−04
CCL3	0.69	1287	1.24 [1.17,1.33]	3.55e−11
EN−RAGE	0.98	1287	1.23 [1.09,1.39]	5.58e−04
FAS	0.35	1287	1.23 [1.11,1.36]	9.15e−05
MPO	0.35	1287	1.23 [1.08,1.39]	1.29e−03
PTX3	0.19	1287	1.20 [1.09,1.31]	1.07e−04
LOX−1	0.67	1287	1.18 [1.05,1.33]	4.59e−03
log2 WBC	0.40	1287	1.18 [1.04,1.34]	8.30e−03
IL−6RA	0.38	1287	1.17 [1.03,1.33]	1.73e−02
CCL4	0.69	1287	1.16 [1.05,1.28]	4.73e−03
TIE2	0.29	1287	1.14 [1.01,1.30]	4.13e−02
SIRT2	0.85	1287	1.14 [1.01,1.29]	3.00e−02
CXCL1	0.70	1287	1.14 [1.01,1.28]	3.83e−02
Gal−3	0.40	1287	1.13 [0.99,1.28]	6.06e−02
Dkk−1	0.50	1287	1.11 [0.98,1.26]	8.81e−02
FS	0.36	1287	1.09 [0.98,1.22]	1.10e−01
PECAM−1	0.33	1287	1.09 [0.96,1.24]	1.80e−01
IL−18	0.58	1287	1.08 [0.95,1.23]	2.34e−01
CASP−8	0.57	1287	1.07 [0.95,1.21]	2.63e−01
HSP−27	0.88	1287	1.07 [0.94,1.20]	3.10e−01
PRL	0.74	1287	1.06 [0.94,1.20]	3.28e−01
IL−16	1.21	1287	1.06 [0.92,1.21]	4.27e−01
PSGL−1	0.19	1287	1.04 [0.92,1.17]	5.22e−01
NEMO	0.68	1287	1.04 [0.91,1.18]	5.77e−01
KLK6	0.48	1287	1.04 [0.91,1.17]	5.77e−01
mAmP	0.93	1287	0.99 [0.87,1.12]	8.46e−01
GAL	0.79	1287	0.99 [0.87,1.12]	8.21e−01
LEP	1.10	1287	0.98 [0.86,1.11]	7.39e−01
log2 LDL cholesterol (mg/dL)	0.46	1287	0.96 [0.85,1.09]	5.01e−01
PAPPA	0.10	1287	0.95 [0.80,1.14]	5.87e−01
PDGF−Subunit−B	0.91	1287	0.94 [0.83,1.07]	3.42e−01
SELE	0.68	1287	0.93 [0.82,1.06]	3.04e−01
log2 HDL cholesterol (mg/dL)	0.38	1287	0.91 [0.80,1.03]	1.42e−01
TRAIL	0.35	1287	0.91 [0.80,1.03]	1.37e−01
log2 triglycerides (mg/dL)	0.70	1287	0.90 [0.79,1.02]	1.04e−01
SRC	1.03	1287	0.89 [0.79,1.01]	7.69e−02
CD40−L	0.35	1287	0.85 [0.71,1.02]	7.36e−02
EGF	1.01	1287	0.85 [0.74,0.97]	1.34e−02
SCF	0.58	1287	0.83 [0.74,0.94]	3.88e−03
log2 hemoglobin (g/dL)	0.16	1287	0.80 [0.71,0.90]	1.79e−04
log2 eGFR CKD−EPI	0.44	1287	0.79 [0.74,0.85]	5.95e−10
TRANCE	0.72	1287	0.75 [0.67,0.85]	2.07e−06

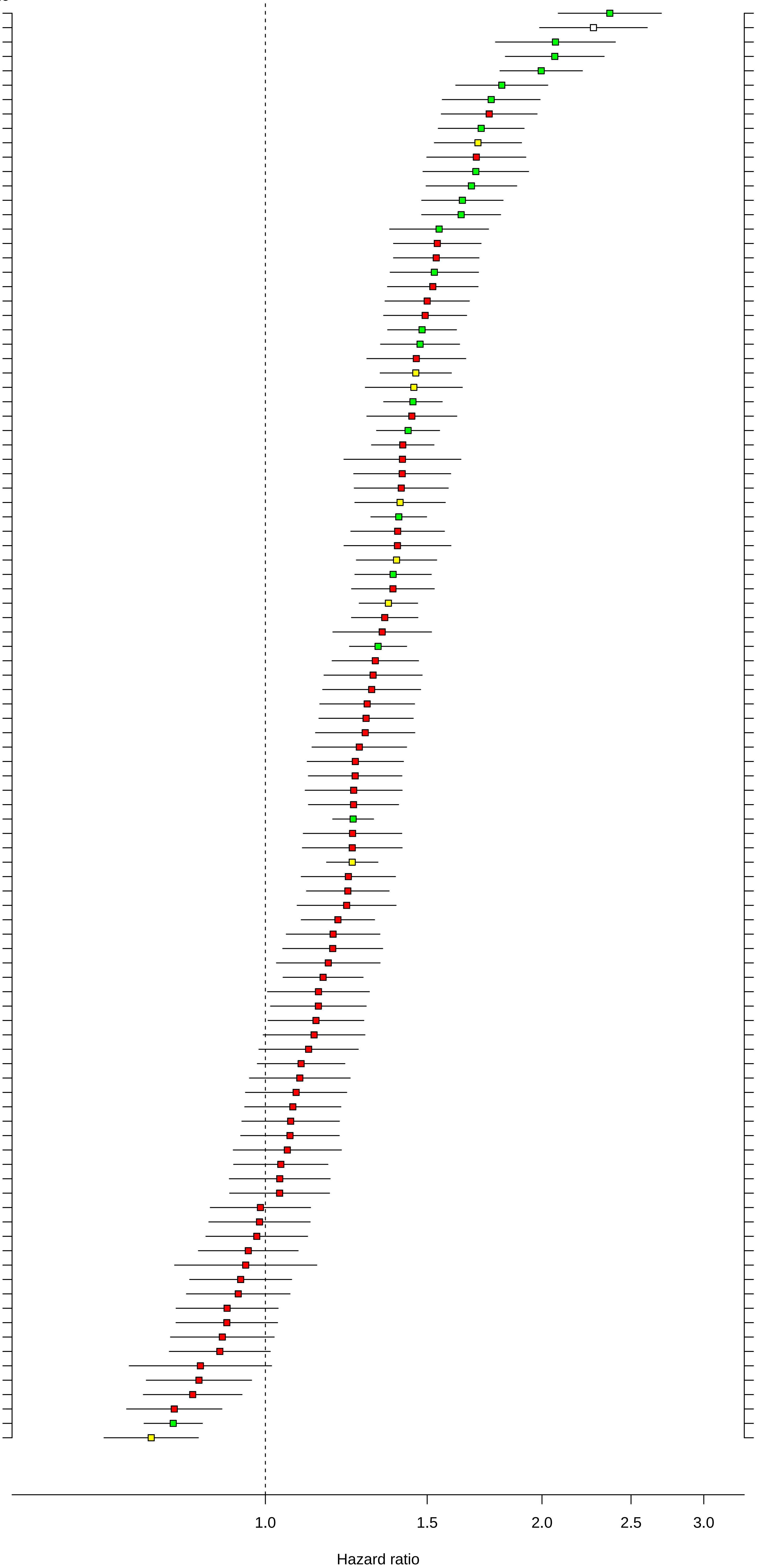

Supplement: S2 Fig — Values are the same as for Fig 1. HR and 95% CI are calculated for increase of 1 SD. Color coding according to the Boruta analysis result: green = confirmed, yellow = tentative, and red = rejected. Variables colored white were not included in the Boruta analysis. CI, confidence interval; CV, cardiovascular; HR, hazard ratio; LURIC, Ludwigshafen Risk and Cardiovascular Health; PEA, proximity extension assay; SD, standard deviation; STABILITY, STabilization of Atherosclerotic plaque By Initiation of darapLadIb TherapY. (PDF) [file pmed.1003513.s010.pdf]
