# Supplementary material for: Plasma proteins associated with cardiovascular death in patients with chronic coronary heart disease: A retrospective study
Source: PLoS Med. 2021 Jan 13;18(1):e1003513. doi: 10.1371/journal.pmed.1003513 (PMC7817029; doi:10.1371/journal.pmed.1003513)
Supplement: Attachment — Submitted filename: Answer to Comments from the Editor and Reviewer.docx [file pmed.1003513.s011.docx]

Re manuscript PMEDICINE-D-20-04160R1: *“Screening of plasma proteins associated with cardiovascular death in patients with chronic coronary heart disease – a retrospective study”.*

**Answers to comments from the Academic Editor and the reviewers-**

**Comments from Academic Editor**

Tone down the causal nature of the observations. There is too much emphasis on 'mechanisms' and I'd say that these are potential mechanisms. In cardiovascular research, there is little evidence to suggest that risk factors differ for primary or secondary prevention. Can they discuss the implications of restricting the population to those with CHD only? Would one expect a different pattern when investigated in a different at-risk group?

Please revise your title according to PLOS Medicine's style. Your title must be nondeclarative and not a question. It should begin with main concept if possible. "Effect of" should be used only if causality can be inferred, i.e., for an RCT. Please place the study design ("A randomized controlled trial," "A retrospective study," "A modelling study," etc.) in the subtitle (ie, after a colon).

**Answer: Title changed to “Screening of plasma proteins associated with cardiovascular death in patients with chronic coronary heart disease – a retrospective study”.**

Abstract- please provide brief participant demographics

**Answer: Demographics have been included in the revised abstract.**

Abstract-please provide 95% CI and p-values when describing association

**Answer: Although very space consuming the HR and 95% CI and p-values from the Cox regression analyses when adjusting for baseline characteristics in the validation cohort have included in the revised abstract.**

Abstract-The last sentence of the methods and findings section should include 2-3 limitations of your study design/methodology

**Answer: Limitations have been included in the revised abstract.**

Abstract conclusions

Please address the study implications without overreaching what can be concluded from the data; the phrase "In this study, we observed ..." may be useful. * Please interpret the study based on the results presented in the abstract, emphasizing what is new without overstating your conclusions. * Please avoid vague statements such as "these results have major implications for policy/clinical care". Mention only specific implications substantiated by the results. * Please avoid assertions of primacy ("We report for the first time....")

**Answer: The conclusions have been adjusted in accordance with the recommendations.**

Author summary

At this stage, we ask that you include a short, non-technical Author Summary of your research to make findings accessible to a wide audience that includes both scientists and non-scientists. The Author Summary should immediately follow the Abstract in your revised manuscript. This text is subject to editorial change and should be distinct from the scientific abstract. Please see our author guidelines for more information: https://journals.plos.org/plosmedicine/s/revising-your-manuscript#loc-author-summary

**Answer: A brief author summary is now included.**

Please use square brackets for references, providing punctuation after a single space. For example xxxx [1,2].

**Answer: References included as requested.**

Methods

Did your study have a prospective protocol or analysis plan? Please state this (either way) early in the Methods section.

**Answer: The prospective statistical analysis plan is stated in the Methods section and included in the supportive material.**

a) If a prospective analysis plan (from your funding proposal, IRB or other ethics committee submission, study protocol, or other planning document written before analyzing the data) was used in designing the study, please include the relevant prospectively written document with your revised manuscript as a Supporting Information file to be published alongside your study, and cite it in the Methods section. A legend for this file should be included at the end of your manuscript.

b) If no such document exists, please make sure that the Methods section transparently describes when analyses were planned, and when/why any data-driven changes to analyses took place.

c) In either case, changes in the analysis-- including those made in response to peer review comments-- should be identified as such in the Methods section of the paper, with rationale.

Please ensure that the study is reported according to the [STROBE] guideline, and include the completed [STROBE or other] checklist as Supporting Information. When completing the checklist, please use section and paragraph numbers, rather than page numbers. Please add the following statement, or similar, to the Methods: "This study is reported as per the Strengthening the Reporting of Observational Studies in Epidemiology (STROBE) guideline (S1 Checklist)."

Please report your study according to the relevant guideline, which can be found here: <http://www.equator-network.org/>

**Answer: The STROBE checklist is included in the submission.**

Please introduce Lp-PLA2   on first view

**Answer: The Lp-PLA2 is introduced as requested in the revised manuscript.**

Please provide participant details for both cohorts- including details of where they were recruited, dates of recruitment and follow up.

**Answer: Participant details are provided.**

Please provide p-values of up to three decimal places throughout the text. Please ensure these are accompanied by 95%CI

**Answer: Three decimal p-values and 95% CI provided in the text**

Page 17 Line 216 suggest replacing “confirmed importance for CV death” to “associated with CVD death” or similar

**Answer: Not changed as “confirmed importance” is the terminology used for significant difference from chance in the Boruta analyses.**

Discussion

Please present and organize the Discussion as follows: a short, clear summary of the article's findings; what the study adds to existing research and where and why the results may differ from previous research; strengths and limitations of the study; implications and next steps for research, clinical practice, and/or public policy; one-paragraph conclusion.

I suggest tempering some of the language in this section, “definitely established as key risk indicators” and “demonstrated to outperform” for example. Since this is an observational study please rephrase to remove causal language

Line 268- please remove the word “causal”

You may wish to rephrase optimal treatment here or explain, that patients were treated with xxx medication. You may wish to include this explicit information in the methods.

**Answer: The text in the discussion has been adjusted to reflect the editor’s comments.**

**Re; Comments from the reviewers:**

**Reviewer #1:** "Profiling of plasma proteins associated with cardiovascular death in patients with chronic coronary heart disease" aims to characterize significant biomarkers with an association to coronary heart disease (CHD). Two cohorts were involved, a derivation cohort consisting of 605 cases with CV death and 2788 non-death cases from the STABILITY trial, and a validation cohort of 245 cases with CV death and 1042 non-death cases from the LURIC study, with mass measurement of protein biomarkers achieved using Proximity Extension Assay (PEA) technology.

The main methodology was based on statistical analysis via the Boruta method on random survival forests (RF), with RF intended to estimate the prognostic importance of variables, and Boruta to determine their significance. Cox regression analyses were also performed. Known prognostic biomarkers such as NTproBNP & cTnT-hs were confirmed as associated with CHD, together with a number of other proteins, for a total of 18 biomarkers of 157 examined.

These findings are interesting in that there appears to be relatively weak consensus on protein biomarker associations with CHD, from prior work. However, while mostly comprehensively described, some points might be clarified, particularly pertaining to data availability and the methodology used:

1. The STABILITY cohort and LURIC cohorts appear to have widely varying follow-up periods (median 3.7 years, and up to 12 years respectively). It might be commented on whether this warrants additional treatment (e.g. short-term vs. long-term CHD mortality)

**Answer: In the LURIC trial the associations using 4 years or 12 years follow-up were similar, although with weaker significances at 4 years because of fewer events. Evaluation of shorter time follow-up was avoided because of low event rates and thereby too low statistical power for reliable multivariate evaluation of multiple biomarkers. This has been commented in in the revised version.**

2. The Pearson/Spearman correlation coefficients for the PEA biomarker measurements (as referenced in Line 117) might be provided if possible in the supplementary.

**Answer:** **This is now included in the Supplement (Table S4a)**

3. The relatively low availability of a large number of biomarkers (Table S2a), together with the minimal availability criteria required to be included in further analysis, might be supported in greater detail. For example, the TSLP biomarker is listed as having 99.5% of values below the lower limit of detection, which appears to imply that it qualifies to be considered for assessment of association with CHD, despite having only 1 in 200 subjects having data available for it. While there is no objection against having such biomarker values reported in principle, the qualification for further analyses might be justified (especially as such values appear to be automatically imputed from Line 179, despite the scant basis for such imputation)

**Answer: We agree that there are some PEA markers with a high percentage of values below LOD. If we had used only Cox-regression as analysis method we would have used a more stringent cut-off for excluding markers, e.g., based on the proportion below LOD. However, since our primary analyses was random Forest we included the opportunity to discriminate between cases and controls also by identifying a measurable value or not. Therefore, we did not use any arbitrary cut-off level for LOD but rather preferred to keep as many proteins as possible in the analyses.**

4. Further on this, certain biomarkers such as BNP (39.3% availability in STABILITY) appear to be presented as significant (BNP is the third top variable, in Figure S1a), despite relatively low availability. It is not immediately evident whether there might be any bias involved towards the subset of patients with detectable BNP, given that this value was not available for nearly 40% of patients. Moreover, is data availability accounted for in determining significance for the statistical analysis? This might be further commented on.

**Answer: The results for BNP seems reliable since we have corresponding results for NT-proBNP (measured with the conventional ROCHE Elecsys Emethod) which has an R2 (squared spearman correlation) > 0.8 with PEA-BNP in STABILITY. The significance of p-values were not adjusted for proportion imputed with LOD, especially as the significances were evaluated both with Random Survival Forest and Cox regression analyses.**

5. On the C-index, while it is stated that "...the incremental discriminative value of each biomarker was illustrated by the C-index" (Line 175), it is not immediately evident as to how this is arrived at. For example, the C-index for NTproBNP is given as 0.802, in Table 2. Does this C-index reflect the performance of the model given all variables (including/excluding NTproBNP?), and if so, does the C-index of Troponin-T reflect the performance give all variables including/excluding Troponin-T, and possibly also including/excluding NTproBNP? Given the relative novelty of the RF-Boruta analysis, it might be appropriate to devote slightly more text to explaining it (possibly in the supplementary material)

**Answer: The C-indices before and after adjustment for clinical variables and the established biomarker levels have been clarified in the footnotes of the Tables of the revised manuscript.**

6. The "By SD" column for Tables 2 & 3/Figures 2 & 4 might be initially defined, together with abbreviations such as those for hazard ratio & confidence interval.

**Answer: Explanations for SD and HR added to the appropriate legends.**

7. The biomarker unadjusted values for HR, CI & C-index (as referenced in Line 171) might also be provided as for the adjusted values in Tables 2 & 3.

**Answer: The unadjusted results are included as figures in the Supplement.**

8. From what could be understood, the derivation STABILITY cohort was first used to develop an RF-Boruta model (and its variable importance analyzed), while the validation LURIC cohort was then used to develop a separate/independent RF-Boruta model, though with input in the form of biomarkers of confirmed importance (Line 216) as determined from the derivation cohort model. The associations for both the derivation and validation cohorts are as presented in Table 3.

However, validation might be more commonly understood as applying a model obtained from the training (derivation) data, to the validation data, and reporting the performance (possibly C-index in this case). This might be considered.

**Answer: We think that our change of terminology to “replication” in the revised manuscript might resolve this matter.**

**Reviewer #2:** The authors Lars Wallentin et al, proposed to reviewer an interesting publication entitled  "Circulating biomarkers are associated with development of coronary heart disease (CHD) and its complications by reflecting pathophysiological pathways and/or organ dysfunction".

They explored the associations between cardiovascular (CV) and inflammatory biomarkers and CV death using proximity extension assays (PEA) in 605 cases with CV death and 2,788 randomly selected non-cases during 3 - 5 years follow-up in the STABILITY trial

They seek for validation of their findings in in the LURIC cohort consisted of 245 cases and 1,042 non-cases during 12 years follow-up.

Authors to conclude that Protein profiles based on clusters of biomarkers reflecting different pathophysiologic mechanisms might be useful for identification of new treatment targets and balancing different treatments in patients with chronic CHD.

While the manuscript is really pleasant to read, there is several points the reviewer which to be seen addressed.

Minor points:

-	Trial registration is given for STABILITY study and not for the LURIC one

**Answer: The LURIC study is an observational study and therefore has no trial registration number.**

-	The methods previously used to measure NTproBNP, cTnT-hs, CRP-hs, IL-6, GDF-15 and Lp-PLA2 should be mentioned

**Answer: As these methods and results previously have been published from this study, we have preferred to only refer to the corresponding publications. “Established biochemical assays for NTproBNP, cTnT-hs, CRP-hs, IL-6, GDF-15, and Lp-PLA_2_ were centrally performed as previously published.(12-17)”**

-	Table 1 in the legend "For BMI, weight, and biomarkers…" this is not relevant as BMI, weight are not presented in this table. However, this remark stands for the Age of the patients

**Answer: The legend is corrected including the specification that “Categorical variables are reported as % (n) whereas continuous are reported by the percentiles 25^th^/50^th^/75^th^.”**

-	Table S3, presentation by percentils 25th/50th/75th are also relevant for the age

**Answer: Corrected as above**

-	Table 1, 2, 3 & 4: a color code should be givent to understand the meaning of the red, yellow, blue and green boxes

**Answer: Unfortunate omission. The following explanation is added: “Color coding according to Boruta analysis result, Green = Confirmed, Yellow = Tentative and Red = Rejected”**

-	Page 23, line 300-303: "The lack of complete replication … relate to many factors e.g…." important to add "handling and age of the biosamples" as the stability  overtime of the biosamples could be critical.

**Answer: Text revised in accordance with the suggestions.**

Major points:

-	The authors detailed within TableS1 the panel from which the measurements have been used for the 27 redundant biomarkers. It is important to have this description as well in the main tables. Furthermore, it is also important to have a comparison of the results obtained on both panels for those 27 proteins present. What are the reproducibility & variability between the two panels while using the same samples?

**Answer: As stated in the manuscript the PEA measurements had acceptable reproducibility with Pearson correlation coefficients 0.80 – 0.97 for 23 and a standard deviation of the difference in levels of 0.18 – 0.31 for 17 of the 27 proteins that were included in both the CVD I and Inflammation panels. These results are presented as the first paragraph in the revised Results section and the tabular presentation of the details has been added to the Supplement (Table S4a)**

| Biomarker | mean.diff | sd.diff | l.diff | u.diff | correlation |
| --- | --- | --- | --- | --- | --- |
| Beta-NGF | -0,82 | 0,53 | -1,86 | 0,21 | 0,62 |
| CASP-8 | -0,79 | 0,4 | -1,58 | 0 | 0,93 |
| CCL20 | -3,38 | 0,28 | -3,94 | -2,82 | 0,97 |
| CCL3 | -1,56 | 0,23 | -2,01 | -1,12 | 0,94 |
| CCL4 | -2,9 | 0,57 | -4,02 | -1,79 | 0,78 |
| CD40 | -0,97 | 0,24 | -1,43 | -0,5 | 0,88 |
| CSF-1 | -3,18 | 0,18 | -3,54 | -2,82 | 0,77 |
| CX3CL1 | -1,11 | 0,24 | -1,58 | -0,65 | 0,87 |
| CXCL1 | -0,75 | 0,37 | -1,48 | -0,02 | 0,96 |
| CXCL6 | -1,54 | 0,32 | -2,16 | -0,92 | 0,96 |
| EN-RAGE | -0,89 | 0,45 | -1,77 | -0,01 | 0,9 |
| FGF-23 | -0,11 | 0,26 | -0,62 | 0,4 | 0,95 |
| HGF | -0,9 | 0,22 | -1,32 | -0,47 | 0,88 |
| IL-18 | -4,79 | 0,23 | -5,24 | -4,33 | 0,93 |
| IL-4 | -0,79 | 0,59 | -1,95 | 0,38 | 0,61 |
| IL-6 | -1,22 | 0,24 | -1,7 | -0,74 | 0,97 |
| IL-8 | -0,7 | 0,27 | -1,22 | -0,17 | 0,96 |
| MCP-1 | -0,5 | 0,22 | -0,92 | -0,07 | 0,9 |
| MMP-1 | 2,35 | 0,36 | 1,64 | 3,05 | 0,95 |
| MMP-10 | -0,03 | 0,26 | -0,54 | 0,48 | 0,91 |
| OPG | -1,09 | 0,2 | -1,49 | -0,7 | 0,87 |
| SCF | 0,07 | 0,18 | -0,28 | 0,43 | 0,93 |
| SIRT2 | -0,02 | 0,68 | -1,36 | 1,31 | 0,94 |
| TNFSF14 | -1,4 | 0,24 | -1,87 | -0,92 | 0,93 |
| TRAIL | -2,57 | 0,18 | -2,92 | -2,22 | 0,86 |
| TRANCE | -0,76 | 0,44 | -1,63 | 0,1 | 0,82 |
| VEGF-A | -0,45 | 0,27 | -0,97 | 0,08 | 0,85 |

-	A comparison of the data obtained with the different methods (conventional & PEA) for the previously used NTproBNP, cTnT-hs, CRP-hs,  IL-6, GDF-15 and Lp-PLA2 would be nice to have. The manuscript suggests an interest in using the multiplex method, thus methods should be compared side by side for the above listed biomarkers.

**Answer: The CRP, cTnT-hs, or Lp-PLA2 are not included in the PEA-analyses. The comparisons between NT-proBNP, IL-6, and GDF-15 are included in the first paragraph of the revised Results section and in the Supplement (Table S4b).**

-	Discussion looks very much like other publications using the PEA or other multiplex technologies (Somalogic, …) even if figure 5 gives a nice resume of the findings. The conclusion on Page 27, line 393 suggesting the" identification of potential treatment targets and balancing different treatments, in patients with CHD" is not enough developed. Even if the topic of the journal is Medicine, important information coming from experimental studies at least on some of the listed biomarkers and all listed pathways could enriched the discussion.

**Answer: The discussion has been adjusted trying to balance the comments from the editor and the reviewers. Speculations on causal inferences have therefore generally been avoided.**

**Reviewer #3:** In this work Wallentin et al. study the association between 157 cardiovascular and inflammatory biomarkers and CV death using proximity extension analysis in patients with stable coronary heart disease.

The derivation cohort consisted of 605 cases (who experienced CV death) and 2788 non-cases (stable CHD patients that survived the 3-5 year follow up).

The validation cohort consisted of 245 cases and 1042 non-cases but the follow up was shorter (1 year).

Biomarker levels were measured using conventional laboratory immunoassays and/or OLINK panels.

28 biomarkers were prognostic in the derivation cohort (most belonged to the CVD panel and only 4 to the inflammation panel).

After validation, a total of 18 biomarkers were found to associate with CV death in both cohorts, of these the strongest predictors were ntProBNP and cTNT-hs.

This is an interesting and data-rich study which highlights the potential clinical utility of multimarker plasma biomarker panels for personalised risk stratification in CHD, yet I have some concerns.

#The abstract focuses on chronic CHD, while the STABILITY and LURIC methods section refers to stable CHD; chronic and stable CHD are not exactly the same thing, so stick to one term.

**Answer: Stable CHD was the terminology used during the STABILITY trial. However, currently chronic CHD is the recommended terminology which therefore has been used in the text of this manuscript.**

#How was stable CHD confirmed in the STABILITY/LRIC studies, did all participants have baseline invasive coronary angiography? (describe this in main text to avoid sending readers to the original papers to find out).

**Answer: The inclusion criteria are further clarified in the revised manuscript.**

#Define CV death and explain how was it ascertained - death certificates, GP records, national statistics office etc?

**Answer: The outcome definitions are clarified in the revised manuscript.**

#Discuss the potential impact of having differential followup duration in the derivation and validation cohorts - consider running a sensitivity analysis on the derivation cohort to check whether the significant biomarkers persist if the follow up duration is whittled to 1year to match that used in the validation cohort.

**Answer: The different follow-up durations have now been commented on in the Limitations section.**

#The author's description of PEA technology in the introduction can be better clarified. It is key to understanding the paper so would be good to explain it at the outset and not later in the discussion.

"Proximity Extension Assay (PEA) technology, allowing simultaneous measurements of 92 proteins in a 1.0 μl plasma sample by PCR amplification of DNA strands from DNA-labeled antibody pairs"

(from OLINK site: A pair of oligonucleotide-labeled antibodies ("probes") are allowed to pair-wise bind to the target protein present in the sample in a homogeneous assay, with no need for washing. When the two probes are in close proximity, a new PCR target sequence is formed by a proximity-dependent DNA polymerization event...the resulting sequence is subsequently detected and quantified using standard real-time PCR.)

**Answer: In the present manuscript the PEA methodology is detailed in the section on “Biochemical analyses”. We think it might be easier to grasp the content of the manuscript if these details remain in this section.**

#The section about reproducibility of assays (ln 117) should be moved to the results section, it is a result.

**Answer: As suggested this section is now presented first in the Results section of the revised manuscript.**

#Did you consider adjusting for socio-economic position in the regression analyses?

**Answer: No**

#You randomised for the administration of darapladib in the regressions, but could you explain to the reader what is the expected effect of darapladib Rx in these patients? Are we to expect lower death rates in darapladib-treated patients?

**Answer: The lack of any significant effect of darapladib is added in the revised manuscript**
